# *Helicobacter pylori* PldA modulates TNFR1-mediated p38 signaling pathways to regulate macrophage responses for its survival

**DOI:** 10.1080/19490976.2024.2409924

**Published:** 2024-10-06

**Authors:** Wei Yang Sit, Mei-Ling Cheng, Tsan-Jan Chen, Chia-Jo Chen, Bo-Nian Chen, Ding-Jun Huang, Pei-Lien Chen, Yun-Ching Chen, Chi-Jen Lo, Deng-Chyang Wu, Wan-Chen Hsieh, Chung-Ting Chang, Ruey-Hwa Chen, Wen-Ching Wang

**Affiliations:** a Institute of Molecular and Cellular Biology & Department of Life Science, National Tsing Hua University, Hsinchu, Taiwan, ROC; b Department of Biomedical Sciences, Chang Gung University, Taoyuan, Taiwan, ROC; c Institute of Biomedical Engineering, National Tsing-Hua University, Hsinchu, Taiwan, ROC; d Division of Gastroenterology, Department of Internal Medicine, Kaohsiung Medical University Hospital, Kaohsiung, Taiwan, ROC; e Institute of Biological Chemistry, Academia Sinica, Taipei, Taiwan, ROC

**Keywords:** Host-pathogen interaction, innate immunity, membrane fluidity, *H. pylori* phospholipase A, TNFR1 signaling

## Abstract

*Helicobacter pylori*, a dominant member of the gastric microbiota was associated with various gastrointestinal diseases and presents a significant challenge due to increasing antibiotic resistance. This study identifies *H. pylori*’s phospholipase A (PldA) as a critical factor in modulating host macrophage responses, facilitating *H. pylori* ‘s evasion of the immune system and persistence. PldA alters membrane lipids through reversible acylation and deacylation, affecting their structure and function. We found that PldA incorporates lysophosphatidylethanolamine into macrophage membranes, disrupting their bilayer structure and impairing TNFR1-mediated p38-MK2 signaling. This disruption results in reduced macrophage autophagy and elevated RIP1-dependent apoptosis, thereby enhancing *H. pylori* survival, a mechanism also observed in multidrug-resistant strains. Pharmacological inhibition of PldA significantly decreases *H. pylori* viability and increases macrophage survival. *In vivo* studies corroborate PldA’s essential role in *H. pylori* persistence and immune cell recruitment. Our findings position PldA as a pivotal element in *H. pylori* pathogenesis through TNFR1-mediated membrane modulation, offering a promising therapeutic target to counteract bacterial resistance.

## GRAPHICAL ABSTRACT

**Figure:**
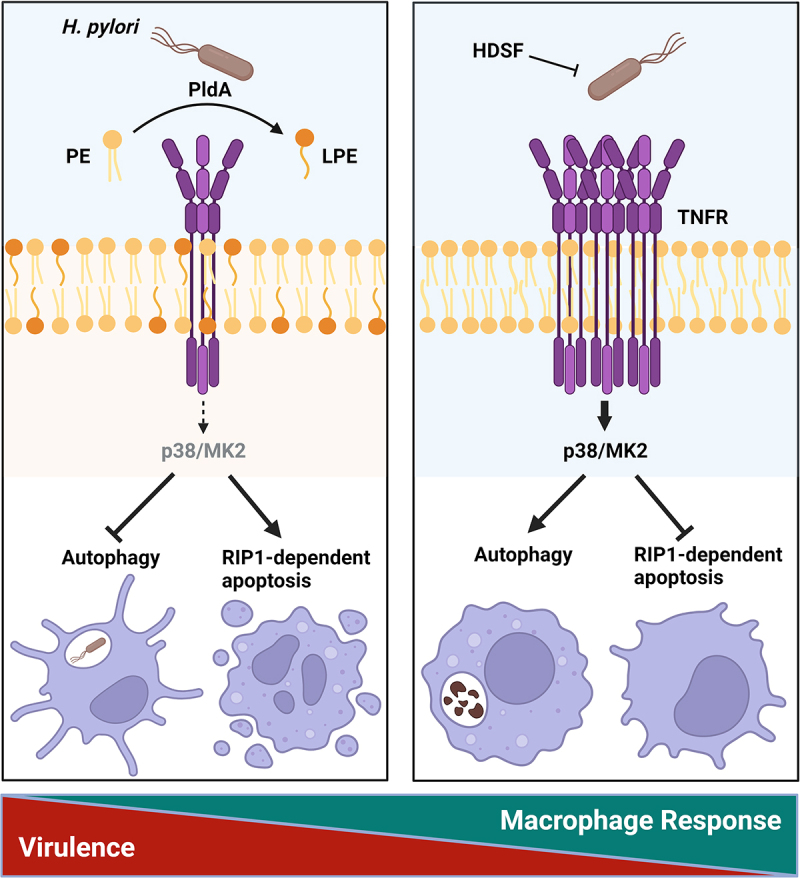

## Introduction

*Helicobacter pylori* is a widespread and persistent pathogen that causes various gastrointestinal diseases, including gastritis, peptic ulcers, and gastric cancer.^1,2^ The prevalence of *H. pylori* infection, combined with the growing concern regarding antibiotic resistance, highlights the complexity of *H. pylori*'s survival strategies within the human body.^3^ Infections with *H. pylori* are characterized by their ability to cause long-lasting inflammation in the stomach.^4^ As a result of this chronic inflammatory response, immune cells infiltrate the area, aiming to combat infection.^5^ Instead of resolving the infection, this response often results in prolonged tissue damage, contributing to more severe clinical sequelae.^6^ *H. pylori*‘s interaction with the human host, particularly its ability to evade the host’s innate immune system, is noteworthy. It has developed unique survival strategies, including the ability to induce apoptosis in monocytes and macrophages, potentially through autophagic pathways.^7–9^ Further complicating *H. pylori*'s interaction with the host immune system is its ability to delay phagocytosis and survive within autophagosomes, a survival tactic that demonstrates *H. pylori*'s adaptive mechanisms to host defenses.^10–12^ This interaction highlights *H. pylori*’s capacity to modulate host immune responses and suggests the complexity of the immune evasion strategies it employs. The failure of the innate immune system to effectively combat *H. pylori* is a critical factor in the pathogen’s persistent colonization and the chronic nature of the infection.

*H. pylori* maintains its persistence and pathogenicity partly due to a complex host-pathogen interplay significantly influenced by its membrane-associated virulence factors. Cytotoxin-associated gene A (CagA) and vacuolating cytotoxin A (VacA) are predominantly found in more virulent type I *H. pylori* strains.^13^ These strains significantly delay phagocytosis, enhancing the bacterium’s ability to evade host immune responses.^10^ In addition, *H. pylori* employs cholesterol-α-glycosyltransferase (CGT) for glycosylation of its membrane.^14^ This process aids in avoiding phagocytosis during infections^15^ and facilitating the delivery of CagA into epithelial cells.^16^

Another key player in this mechanism is phospholipase A (PldA), which acylates cholesteryl glucosides (CGs) to form acylated forms (CAGs), enhancing the adhesion of *H. pylori* to epithelial cells.^17^ Furthermore, studies have demonstrated that *H. pylori* can be detected in the double membrane of the autophagosomes^18^ and is capable of invading and persisting not only in epithelial cells but as well as phagocytic cells.^19–21^ There is also evidence that *H. pylori* directly interacts with macrophages *in vivo*,^22,23^ suggesting that autophagy is a critical host defense mechanism against *H. pylori* infection. While several studies have uncovered how this pathogen exploits the host’s epithelial cell autophagy machinery, little is known about the mechanisms and the critical virulent factors involved in the interaction between *H. pylori* and immune cell autophagy. PldA also exhibits phospholipase and hemolytic activity.^24,25^ An *H. pylori* mutant lacking this enzyme fails to colonize the gastric mucosa effectively in animal models.^24^ Notably, PldA is not unique to *H. pylori*; it is a highly conserved transmembrane serine hydrolase found in various gram-negative bacteria.^26,27^ For instance, *Escherichia coli* PldA (OMPLA) exhibits a wide range of phospholipase specificity and activities, indicating the enzyme’s broad biological significance.^26^

Despite significant progress in understanding the pathogenesis of *H. pylori* and its interactions with the host immune system, the full spectrum of its molecular mechanisms and impacts is not yet fully understood. A critical area of research is the interaction of membrane-associated virulence factors with host innate immunity. The objective of this study is to investigate how *H. pylori*'s membrane-associated virulence factors affect macrophage responses. Our findings suggest that PldA is a unique factor that affects the autophagic responses of macrophages during infection. We found that *H. pylori* PldA increases the levels of lysophosphatidylethanolamine (LPE), which causes changes in membrane integrity and disrupts TNFR1 receptor clustering. Consequently, the activation of the p38-MK2 pathway is reduced, leading to decreased autophagy and increased RIP1-induced apoptosis. This study reveals a previously unknown mechanism by which PldA interferes with the host’s immune response, contributes to its virulence, and highlights its potential as a promising target for combating drug-resistant Gram-negative bacteria. The findings demonstrate that using a PldA inhibitor may be an effective treatment for reducing the intracellular survival of multidrug-resistant *H. pylori* strains.

## Results

### Effect of H. pylori virulence factors on macrophage recruitment and autophagy modulation

**Figure 1.**
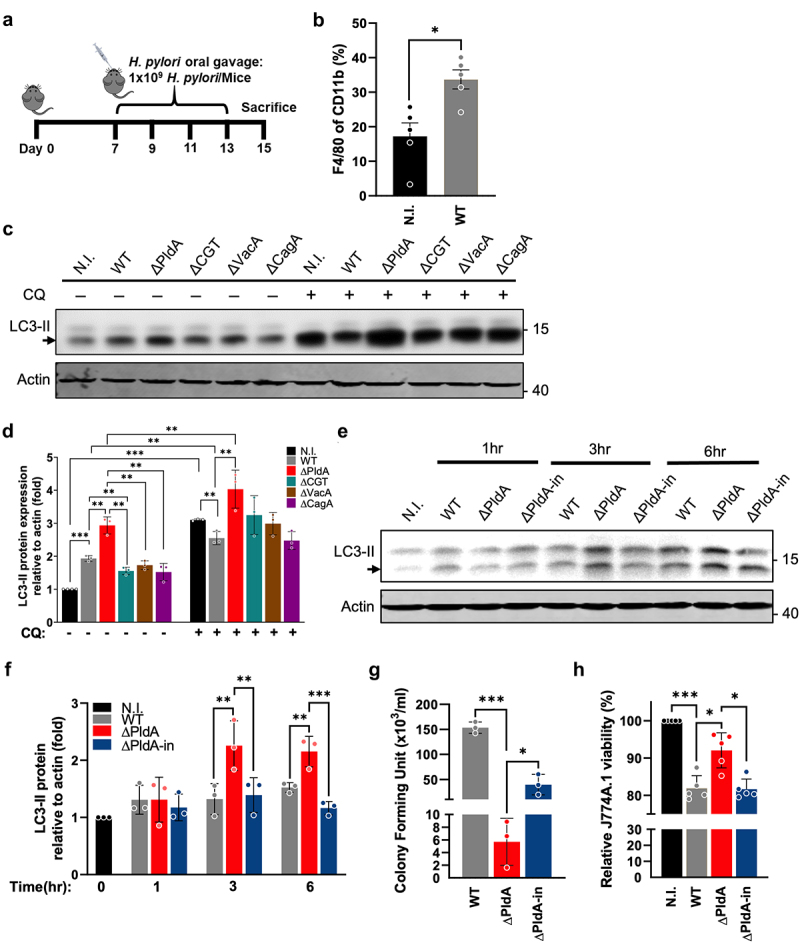
Effect of *H. pylori* virulence factors on macrophage recruitment and autophagy modulation. (a) C57BL/6 mice received orogastric inoculations every 2 days with WT *H. pylori* (1 × 10^9^ CFU), resulting in 4 total inoculations. Infiltration levels of F4/80+/CD11b+ macrophages (b) in gastric tissues following the regimen described in (a). (c) J744A.1 cells were uninfected (N.I.) or infected with WT, isogenic mutant ΔPldA, *capJ*-knockout (ΔCapJ), *vacA*-knockout (ΔVacA), or *cagA*-knockout (ΔCagA) at a MOI of 100 at 37°C and under 5% CO_2_ for 3 hr. Chloroquine (CQ) was added to the cells at a concentration of 10 μM before infection with different strains of *H. pylori*. the expression levels of LC3-II were detected by Western blotting analysis. Actin was used as an internal control. (d) quantification of the ratio of LC3-II to actin in (c). (e) J744A.1 cells were infected with WT, Δ PldA, or ΔPldA-in at a MOI of 100 for 1, 3, and 6 hr, respectively. LC3-II expression was determined by Western blot analysis. Actin was used as an internal control. (f) quantification of the ratio of LC3-II to actin in (e). Each value represents the mean ± SD from 3 independent experiments in (d) and (f). (g to h) J744A.1 cells were infected with WT, ΔPldA, or ΔPldA-in at a MOI of 100 for 3 hr. Gentamicin CFU assays were used to determine the viability of internalized *H. pylori* (g). J744A.1 viability (uninfected or infected with WT, ΔPldA, or ΔPldA-in) was assessed using trypan blue exclusion assays. Results are presented relative to the viability of uninfected macrophage (h). Each value represents the mean ± SD from 3 (g) or 5 (h) independent experiments. (b) data points represent individual mice, with bars indicating mean ± SEM collated from 3 separate experiments. Statistical analysis involved the Mann-Whitney test and subsequent Dunn’s post hoc test. (d, f to h) statistical significance (α = 0.05) was calculated using a two-tailed unpaired Student’s *t*-test. *p < .05, **p < .01, **p < .001.

To investigate *H. pylori*’s influence on host immune responses during the early phase of colonization, we conducted *in* *vivo* experiments using C57BL/6 mice infected with wild-type *H. pylori* (WT *H. pylori*) (Figure 1a). This infection resulted in a slight increase in CD45+ leukocyte infiltration, although this was not statistically significant (Fig. S1A). Notably, *H. pylori*-infected mice exhibited significant macrophage infiltration in gastric tissues (Figure 1b). However, the numbers of neutrophils (Fig. S1B) and CD4+ T cells (Fig. S1C) remained unchanged in the *H. pylori*-infected group compared to the non-infected control group, indicating specific recruitment of macrophages in response to *H. pylori* infection.

To complement these *in vivo* findings, we assessed immune cell population alterations in patients with and without *H. pylori* infection based on the microarray data set (Accession Number: E-MTAB-8889).^28^ For this analysis, we excluded individuals with both *H. pylori* infection and intestinal metaplasia development, and those without *H. pylori* infection but later developed gastritis (Table S1). Employing Cibersortx, a gene expression-based tool for estimating immune cell abundance.^29^ Our analysis indicated a significant elevation in the population of M1 macrophages in patients infected with *H. pylori* (Fig. S1D). While increases in M0 and M2 macrophages were observed, these changes were not statistically significant (Fig. S1E). In line with our *in vivo* data, these findings directed our focus toward macrophages for further detailed study.

We next sought to evaluate the specific virulence factors associated with the cell membrane to understand the mechanisms behind *H. pylori* persistence in macrophages. Since autophagy plays a key role in counteracting *H. pylori* infection,^12^ we investigated the effects of *H. pylori*’s principal virulence factors, including CGT, VacA, and CagA, on macrophage autophagy. We infected J774A.1 cells with either WT *H. pylori* strain 26695 or its isogenic knockout mutants (∆CGT, ∆VacA, or ∆CagA),^16,30^ and autophagy levels were assessed using the LC3 marker.^31^ In addition, we evaluated PldA, which is important in bacterial adherence and colonization. ∆PldA and a complemented PldA strain (∆PldA-in) were produced and validated (Fig. S2A). Thin-layer chromatography (TLC) was employed to confirm the enzymatic activity of PldA (Fig. S2A).

We found that cells infected with the ∆PldA strain exhibited significantly elevated LC3-II levels (as indicated by the lower band) compared to other strains, indicating PldA as a key factor in autophagy regulation (Figures 1c,d). This was further substantiated when cells were treated with chloroquine (CQ), a lysosomotropic agent that inhibits the fusion of autophagosome-lysosome,^32^ commonly used to investigate the total levels of autophagic induction by preventing the degradation of autophagosomes.^33^ In CQ-treated cells, LC3-II levels were reduced in WT, ΔCGT, ΔVacA, and ΔCagA infections compared to CQ-treated uninfected controls, indicating a complex interplay where specific virulence factors modulate autophagy flux (Figures 1c, d). Interestingly, in CQ-treated ∆PldA infections, LC3-II levels were significantly increased compared to WT, suggesting PldA’s potential role in inhibiting autophagy.

Time-course analysis of LC3-II levels in infected cells (WT, ΔPldA, or ΔPldA-in) post-infection (Figures 1e, f) further confirmed that, at 3 and 6 hr post-infection, ∆PldA-infected cells had significantly higher LC3-II levels than WT-infected cells. The reintroduction of PldA in the ∆PldA-in strain significantly reduced this increase, underlining the unique role of PldA in the autophagic response during *H. pylori* infection in macrophages.

The expression of LAMP1, a marker of lysosomes, was measured during infection. The level of LAMP1 increased during ΔPldA infections compared to WT and ΔPldA-in infections (Fig. S2B), suggesting autophagosomes in ΔPldA infection are directed toward the autolysosomal pathway. To evaluate PldA’s influence on the survival of internalized bacteria, we conducted a gentamicin assay. We noted a significantly diminished bacterial survival rate in ∆PldA infections compared to both WT and ∆PldA-in infections (Figure 1g). This was further supported by a qPCR assay (Fig. S2C). Total bacterial levels were quantified, revealing a significant decline in ∆PldA infections compared to WT or ∆PldA-in infections (Fig. S2D). Adherence assays indicated no significant differences in adherence levels between WT and ∆PldA infections over time (Fig. S2E).

Additionally, we evaluated the effect of PldA on macrophage viability using a trypan blue exclusion test.^34^ There was a significant reduction of cell viability in WT-infected cells relative to the uninfected control and those infected with ∆PldA (Figure 1h). The introduction of the ∆PldA-in infection restored cell viability to levels comparable to the WT infection (Figure 1h). Taken together, these results suggest the significance of PldA in regulating autophagy and cytotoxicity, as well as its influence in promoting bacterial persistence within macrophages.

### Lipidomic characterization of H. pylori PldA’s effects on phospholipids in infected and uninfected macrophages

Using mass spectrometry-based lipidomics, we investigated PldA’s impact on phospholipid composition in uninfected and infected macrophages (WT, ΔPldA, or ΔPldA-in). A heat map of altered metabolites with a false discovery rate (FDR)-corrected *q*-value <0.05 by Ward clustering analysis revealed that as compared to infected macrophages, uninfected macrophages exhibited lower abundances of most lysophospholipids and higher abundances of most phospholipids (Fig. S3A). Significant enrichment of lysophospholipids, including LPE and lysophosphatidylcholine (LPC) species, was observed in both WT- and ΔPldA-in-infected macrophages (Fig. S3A).

**Figure 2.**
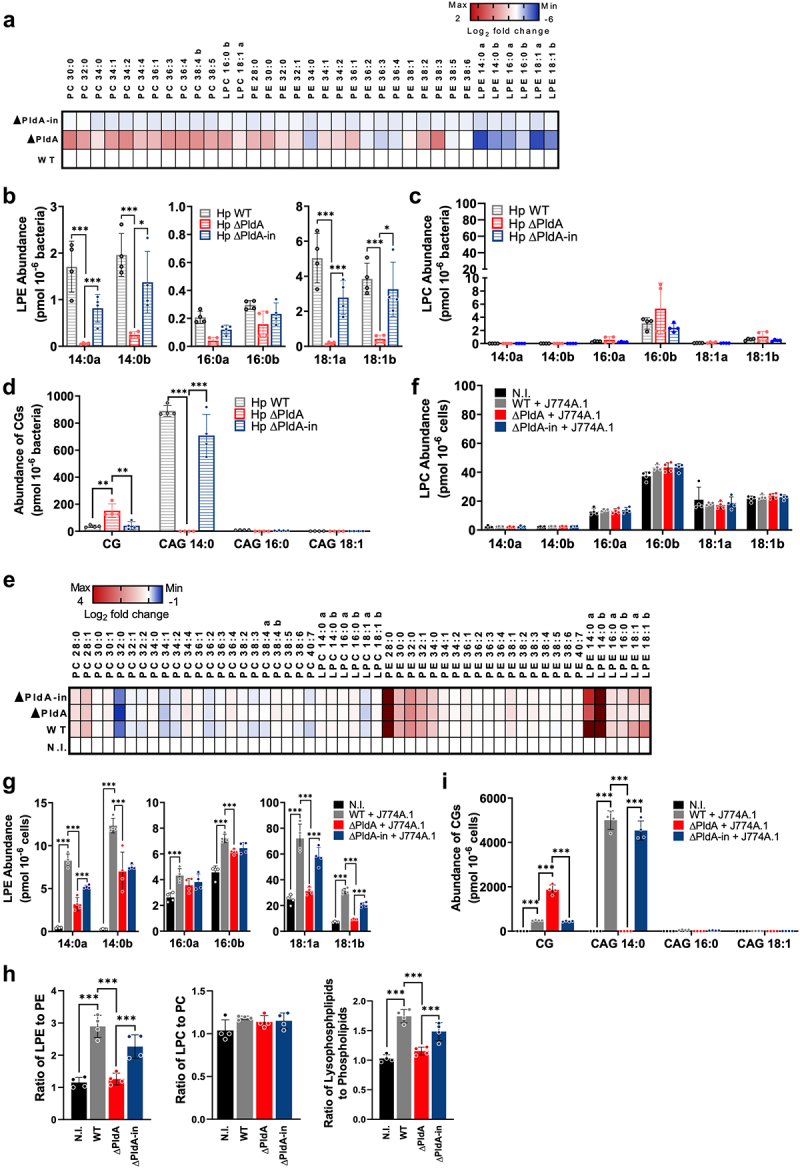
*H. pylori* PldA affects lipid composition. (a) heatmap of lipid changes in *H. pylori* (WT, ∆PldA, and ∆PldA-in). Color scale represents the log_2_ fold change of lipid abundance relative to WT. 4 replicates for each group were performed. (b to d) the abundance of LPEs (b), and LPCs (c), and cholesteryl derivatives (d) (pmol/million bacteria) in *H. pylori*. The data presented are means ± standard deviations from 4 independent experiments. A two-tailed unpaired Student’s *t*-test was used to determine statistical significance (α = 0.05). (e) heatmap of lipid changes in uninfected (N.I.) and infected J774A.1 (WT, ∆PldA, and ∆PldA-in). Cells were infected with WT, ∆PldA, or ∆PldA-in at a MOI of 100 for 3 hr. 4 replicates for each group were performed. Color scale represents the log_2_ fold change of lipid abundance relative to WT. The abundance of LPCs (f), LPEs (g), and cholesteryl derivatives (i) (pmol/million cells). (h) ratio of LPE/PE (left panel), LPC/PC (middle panel) and lysophospholipids/phospholipids (right panel) changes in uninfected (N.I.) and infected J774A.1 (WT, ∆PldA, and ∆PldA-in). The data presented are means ± standard deviations from 4 independent experiments. A two-tailed unpaired Student’s *t*-test was used to determine statistical significance (α = 0.05). A phospholipid is labeled b when it is the (sn)-1 isomer and a when it is the (sn)-2 isomer. *p < .05, **p < .01, **p < .001.

To further examine these changes, we performed a targeted analysis through liquid chromatography coupled with tandem mass spectrometry. We quantitatively compared the levels of selected species of phosphatidylcholine (PC) and phosphatidylethanolamine (PE), LPC, LPE, and cholesterol derivatives (CG, CAG(14:0), CAG(16:0), and CAG(18:1)) in the *H. pylori* alone group (WT, ΔPldA, and ΔPldA-in). The isomers of (Sn-1) and (Sn-2) LPE (16:0) were well separated, identified, and quantified (Fig. S3B). We noted a significant difference in lipid profiles between WT, ∆PldA, and ∆PldA-in (Figure 2a). Compared to WT and ∆PldA-in, all LPE species were significantly reduced in ∆PldA (Figure 2b). WT exhibited significantly higher levels of both Sn-1 and Sn-2 LPE species than ∆PldA, indicating that PldA is capable of cleaving both (Sn)-1 and (Sn)-2 acyl chains of LPE. Conversely, no significant differences in the levels of LPC species were observed between different *H. pylori* strains (Figure 2c). For cholesteryl derivatives, an increase in CG was observed in ΔPldA (Figure 2d). Moreover, WT and ∆PldA-in, but not ∆PldA, showed high levels of CAG(14:0). These results suggest that *H. pylori* PldA plays dual functions in producing LPE and CAG (14:0) species.

We next quantified the abundance of selected metabolites in uninfected and infected macrophages, confirming our initial heat map observations (Figures 2e, h). Specifically, we detected increased levels of LPE species in infected macrophages, particularly with WT and ΔPldA-in infections (Figures 2e, f). On the other hand, there were no significant differences in the levels of LPC species across groups (WT, ΔPldA, and ΔPldA-in) (Figure 2g). The ratios of LPE/PE and lysophospholipids/phospholipids were significantly elevated in WT and ΔPldA-in infected macrophages compared to ΔPldA-infected ones (Figure 2h). A comparison of the LPE(18:1)/LPE(14:0) ratio between the *H. pylori*-alone and the *H. pylori*-infection groups (Figures 2b, g) revealed a substantial increase in the WT *H. pylori*-infection group, particularly (Sn)-2 LPE(18:1)/(Sn)-2 LPE(14:0) (2.9 vs. 8.0).

We assessed uninfected or infected macrophages for the presence of CG and CAG. No CAG signals were detected in macrophages infected with ΔPldA, while an increase in CG levels was observed compared to WT- or ΔPldA-infected macrophages (Figure 2i). A significant decrease in the CAG(14:0)/CG ratio was observed in the group with *H. pylori*-infection (WT or ΔPldA-in) compared to the group with *H. pylori*-alone (Figures 2d, **i** and Fig. S3C).

A lipidomic time course analysis was conducted to evaluate that *H. pylori* PldA targets macrophage membrane PE species as substrates. Our results showed a time-dependent increase in the levels of various LPE species (14:0a, 14:0b, 16:0b, 18:1b) (Fig. S4A‒C) upon infection with *H. pylori* harboring PldA. The levels of LPE species (16:0b and 18:1b) were noted to remain relatively constant throughout the time course experiment in non-infected cells and ΔPldA-infected macrophages, suggesting that the observed LPE increase of 16:0b and 18:1b is specifically due to PldA activity.

To further demonstrate that PldA targets PE of the macrophage plasma membrane, we labeled the macrophage membrane with PED-A1, an Sn-1-specific fluorogenic PE that emits a red fluorescent signal when PE is cleaved to form LPE. The results showed that the presence of PldA in *H. pylori* resulted in significantly higher levels of red fluorescent signal compared to those infected with ΔPldA *H. pylori* (Fig. S4D). This indicates that *H. pylori* PldA directly cleaves macrophage membrane PE, leading to increased LPE levels rather than introducing LPE from the bacteria. These observations align with our lipidomics data in Figures 2 and S4, substantiating that *H. pylori* hijacks the host macrophage membrane by altering its composition through PldA activity.

These findings suggest that PldA-mediated cleavage of macrophage membrane PE leads to the production of LPE species in infected macrophages, potentially influencing host membrane dynamics and signaling pathways.

### H. pylori PldA suppresses the p38-MK2 signaling pathway in macrophages

**Figure 3.**
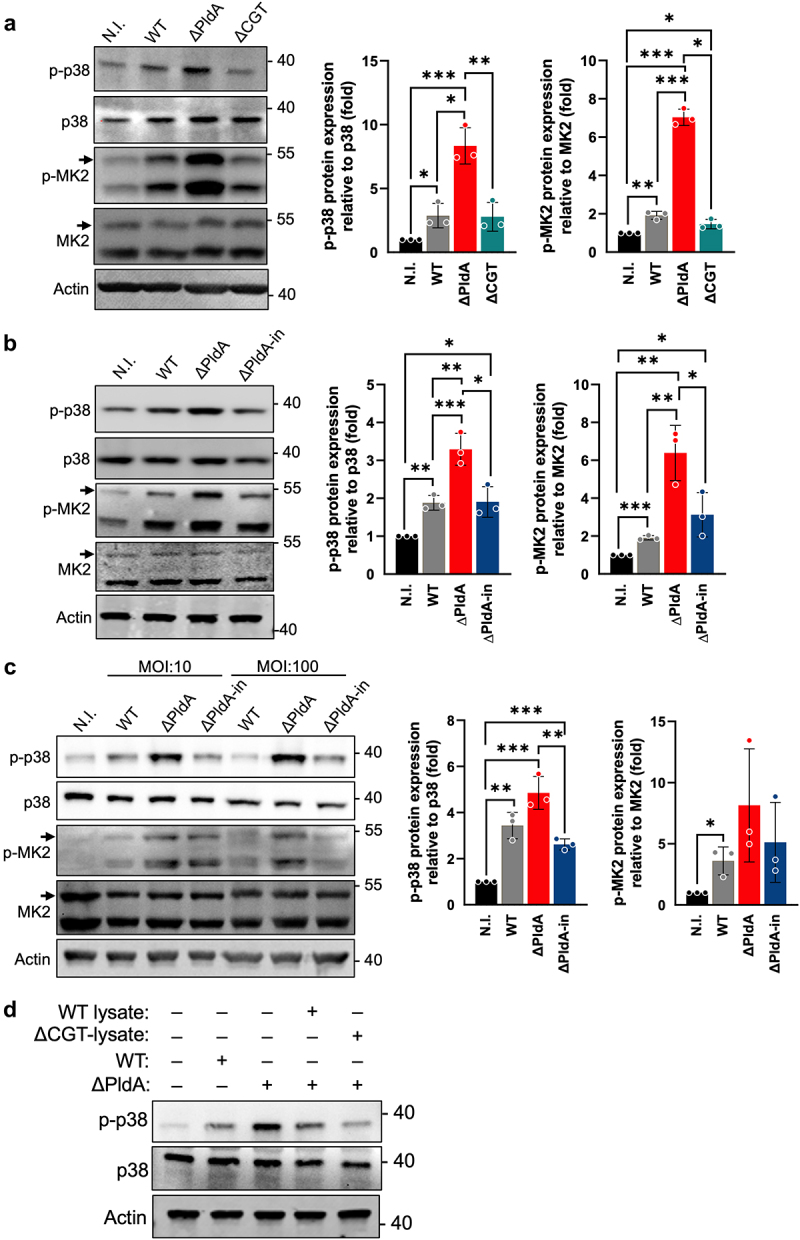
*H. pylori* PldA reduces the activation of p38-MK2 signaling in macrophages. (a to d) the presence of PldA reduces the activation of p38-MK2 signaling in *H. pylori*-infected macrophages. The p38, p-p38, MK2, and p-MK2 levels in uninfected (N.I.) or *H. pylori*-infected J774A.1 (WT, ∆PldA, and ∆CGT) (a), or (WT, ∆PldA, ∆PldA-in) (b) at a MOI of 100 for 3 hr were detected by immunoblotting and the quantified ratio of p-p38 to p38 and pMK2 to MK2 were displayed as the middle and the right panel. The levels of p38 and p-p38 in uninfected (N.I.) or *H. pylori*-infected J774A.1 (WT or ∆PldA) without or with crude lysates from WT or ∆CGT were detected by immunoblotting (c). The p38, p-p38, MK2, and p-MK2 levels in uninfected (N.I.) or *H. pylori*-infected J774A.1 (WT, ∆PldA, ∆PldA-in) at a MOI of 10 or 100 for 3 hr were detected by immunoblotting (d). Actin was used as an internal control. This data is representative of 3 independent experiments and presented as mean ± standard deviations. A two-tailed unpaired Student’s *t*-test was used to determine statistical significance (α = 0.05). *p < .05, **p < .01, **p < .001.

The increased levels of LPE and lysophospholipids in infected macrophages point to potential membrane disruption, which might impact subsequent signaling pathways.^35,36^ By employing micro-western array analysis, we explored the regulation of signaling pathways in macrophages infected by WT or ΔPldA. We observed differential signaling through multiple pathways (GSK3β, PDK1, cJUN, MEK3, and p38) (Fig. S5A). Notably, the p38 MAPK and JNK pathway was particularly evident in cells infected with the WT strain (Fig. S5A). To further clarify the roles of PldA and CGT, two cholesterol glycosylating enzymes, we carried out a western blot analysis. This analysis revealed a distinct increase in the phosphorylation of p38 (p-p38) in macrophages infected with the ΔPldA strains, in contrast to those infected with WT or ΔCGT strains (Figure 3a). Additionally, our findings showed that the JNK signaling pathway remained largely unaffected by PldA, as demonstrated by our Western blot results (Fig. S5B). This increase was further substantiated by enhanced signals for phosphorylated MK2 (as indicated by the upper band), a downstream target of p38 (Figure 3a). Importantly, the elevated p38 activation observed in ΔPldA infection was effectively reversed with ΔPldA-in infection (Figure 3b).

Given the importance of the enzymatic activity of PldA in modulating the macrophage p38 MAPK response, we next investigated whether this effect persists under conditions that more closely mimic *in vivo H. pylori* infection by using a lower MOI. As anticipated, even at a reduced MOI, *H. pylori* infection continued to inhibit p38 MAPK activation in macrophages infected with either WT or ΔPldA-in *H. pylori* (Figure 3c).

We prepared lysates containing PldA from WT or ΔCGT strains. Both lysates effectively reduced p38 activation caused by ΔPldA infection (Figure 3d). When infected macrophages (WT, ΔPldA, or ΔPldA-in) were treated with BIRB796 (a p38 inhibitor), p38-MK2 activation signals were essentially neutralized to levels similar to control cells (Fig. S5C). Conversely, cells infected with ΔPldA and WT showed similar p38 activation after anisomycin (a p38 activator) treatment (Fig. S5D). This together suggests that *H. pylori* PldA targets the membrane-mediated p38-MK2 pathways in macrophages.

### PldA inhibits macrophage autophagy and p38-MK2-dependent beclin 1 (S90) activation

**Figure 4.**
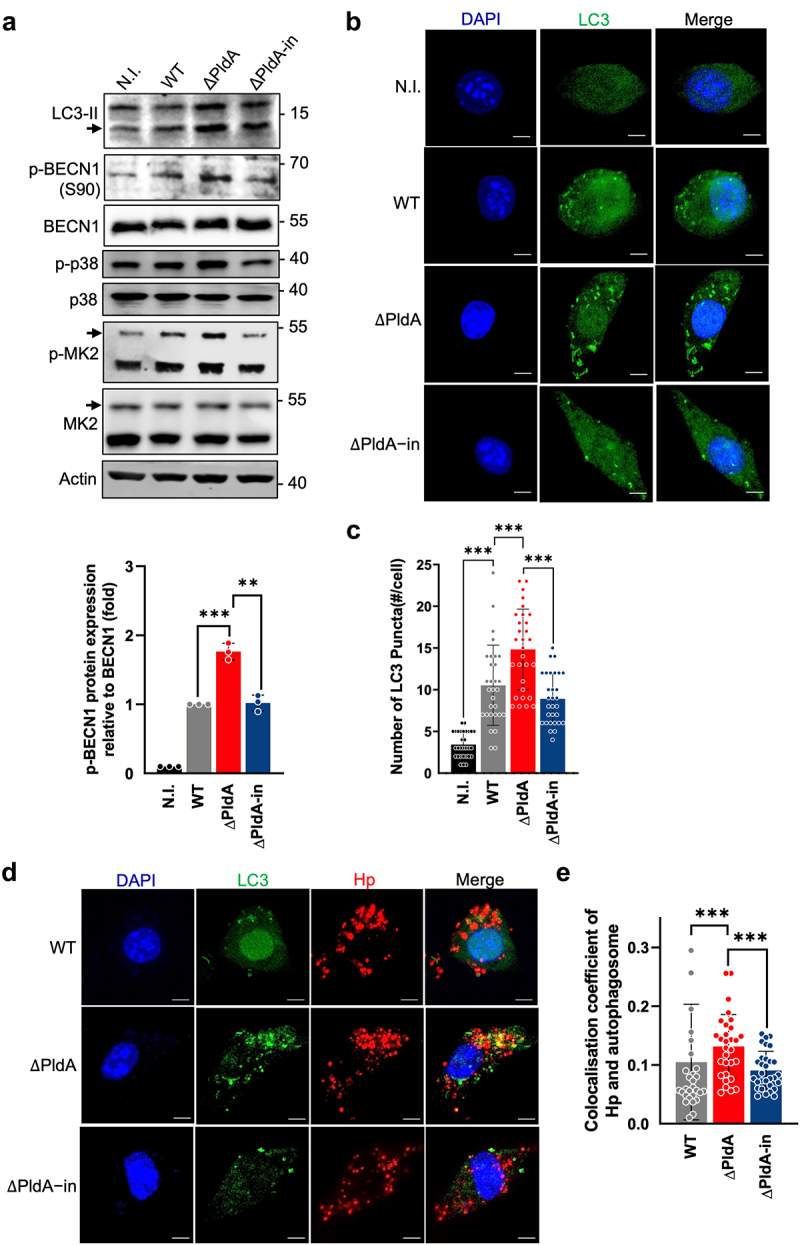
PldA suppresses autophagosome formation in infected macrophages through p38-MK2 signaling. (a) the presence of PldA reduces the phosphorylation of beclin-S90 via the p38-MK2 signaling pathway in *H. pylori*-infected macrophages. LC3-II, p-BENC1 (S90), BECN-1, p-p38, p38, p-MK2, and MK2 levels in uninfected (N.I.) or *H. pylori*-infected J774A.1 (WT, ∆PldA, and ∆PldA-in) at a MOI of 100 for 3 hr were detected by immunoblotting and the quantification of p-BENC1 (S90) to BECN-1 were displayed as the lower panel. Actin was used as an internal control. This data is representative of 3 independent experiments. (b) immunofluorescence staining of LC3-II (green) in uninfected (N.I.) or *H. pylori*-infected J774A.1 (WT, ∆PldA, and ∆PldA-in) cells at a MOI of 100 for 3 hr. Nuclei were stained with DAPI (blue). (c) quantification of the LC3-II puncta formation in (b). (d) immunofluorescence staining of LC3-II (green) and *H. pylori* (red) in uninfected (N.I.) or *H. pylori*-infected J774A.1 (WT, ∆PldA, and ∆PldA-in) cells at a MOI of 100 for 3 hr. (e) colocalization analysis of LC3-II and *H. pylori* in (d). The data in (c) and (e) represent the mean ± SD. Statistical significance was calculated using a two-tailed unpaired Student’s *t*-test *n* = 30. The scale bar represents 5 μm. *p < .05, **p < .01, **p < .001.

The pathway of p38-MK2 plays a crucial role in regulating the response to stress caused by external signals.^37^ We evaluated the phosphorylation of beclin 1 at Ser 90 (p-BECN1 (S90)), a crucial initiation stage of autophagy,^38^ and examined the possible interaction with the p38-MK2 signaling pathway. We noted that infection with ΔPldA increased p-BECN1 (S90) levels compared to infection with WT (Figure 4a). This was accompanied by the increased signals of LC3-II, p-p38 and p-MK2 in infection with ΔPldA (Figure 4a). When infected with ΔPldA-in, the effects were restored (Figure 4a). Using confocal microscopy, ΔPldA infection showed significantly more LC3-II puncta than WT or ΔPldA-in infection (Figures 4b, c). Additionally, we observed a higher degree of colocalization between LC3-II puncta and bacteria following infection with ΔPldA than either WT or ΔPldA-in infection (Figures 4d, e).

After infection with ΔPldA, inhibition of p38 with BIRB796 (100 nM) resulted in a marked decrease in both p-BECN1 (S90) and LC3-II levels, compared to WT or ΔPldA-in infection (Fig. S6A). Furthermore, BIRB796 treatment significantly reduced the formation of LC3 puncta in either WT or ΔPldA infection (Fig. S6B and S6C) and decreased the colocalization of bacterial cells with the autophagosomes (Fig. S6D and S6E). In addition, BIRB796 treatment significantly increased bacterial *16s rRNA* levels in infected macrophages (Fig. S6F), indicating higher intracellular persistence of *H. pylori*. These findings suggest that PldA inhibits the formation of autophagosomes through the p38-MK2-beclin 1 pathway, which enhances *H. pylori*'s survival in infected macrophages.

### PldA disrupts TNFR1-mediated p38-MK2 signaling in macrophages

**Figure 5.**
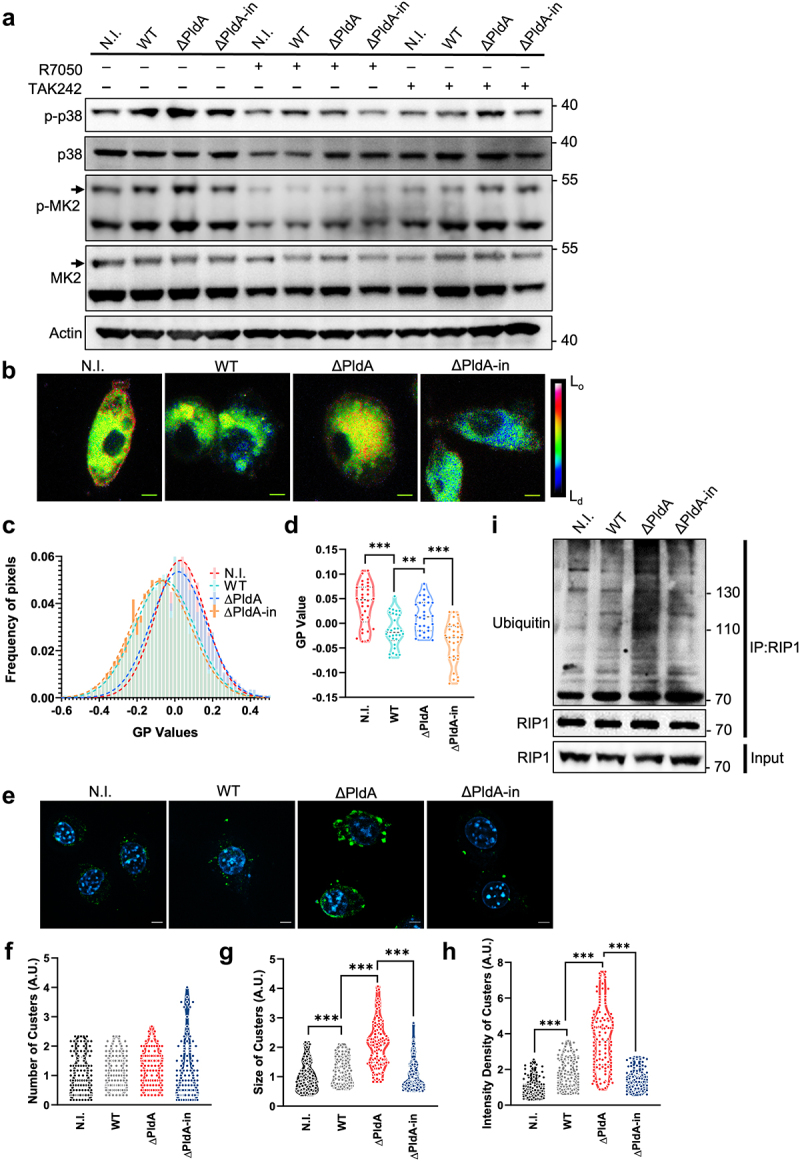
Effect of TNFR1 inhibition or presence of PldA on membrane fluidity, TNFR1 clustering, and RIP1 ubiquitination. (a) J744A.1 was pretreated with DMSO, R7050 (10 μM) or TAK242 (10 μM) for 30 min, followed by non-infection or infection with various *H. pylori* strains (WT, ∆PldA, or ∆PldA-in) at a MOI of 100 for 3 hr. The p-p38, p38, p-MK2 and MK2 levels were detected by immunoblotting analysis. Actin was used as the internal control. (b) A Laurdan imaging analysis of the membrane lipid order in uninfected and infected J744A.1. The general polarisation (GP) value indicates the degree of fluidity in the plasma membrane. The GP images are presented as merged means of intensity and rainbow RGB pseudocolored images. Red indicates less membrane dynamic, while blue indicates high membrane dynamic. Scale bar, 5 μm. (c) GP value distribution in (b). The infection of macrophages with WT and ΔPldA strains resulted in a reduction in the order of the plasma membrane, as indicated by a lower GP relative to ΔPldA-infected J774A.1. (d) The statistical results for the GP values in individual cells are presented. *n* = 30. A two-sided, unpaired Student’s *t*-test was used for these analyses. (e) Immunofluorescence staining of TNFR1 (green) in uninfected (N.I.) or *H. pylori*-infected J774A.1 (WT, ΔPldA, and ΔPldA-in) at a MOI of 100 for 3 hr. Nuclei were stained with DAPI (blue). (f to h) quantification of TNFR1 cluster formation (f), cluster size (g), and intensity density (h) in (e). Statistical analysis was conducted on individual cells. *n* = 100. A two-sided unpaired Student’s *t*-test was used for analysis. Scale bar, 5 μm (I) Co-immunoprecipitation with anti-RIP1 from lysates was carried out to detect the presence of ubiquitinated forms of RIP1. This data is representative of 2 independent experiments. *p < .05, **p < .01, **p < .001.

TNFR1 and TLR4 play critical roles in the regulation of p38-MK2 signaling in response to TNF and bacterial infections.^37,39^ We conducted an experiment to determine whether a TNFR1 antagonist treatment, referred to as R7050, would have an effect. Interestingly, the R7050 treatment dramatically diminished the signals for p-p38 and p-MK2 in infected macrophages (WT, ∆PldA, and ∆PldA-in) (Figure 5a). We investigated the involvement of TLR4 by treating macrophages with TAK242, a specific TLR4 inhibitor, which resulted in a weaker effect (Figure 5a).

We next investigated whether an increase in lysophospholipids from PldA’s activity affects membrane bilayer structure since changes in membrane lipid composition can affect receptor activation.^35^ The plasma membrane ordered phase was determined using the fluorescent probe Laurdan^40^ in uninfected and infected macrophages. Using Laurdan staining to measure membrane order in uninfected macrophages by quantifying the difference between the emission spectrums of ordered and disordered phases, we found that there is a significant decrease in plasma membrane order was observed after WT or ∆PldA-in infection compared to ∆PldA infection (Figures 5b, d), indicating that increased lysophospholipids content reduces membrane rigidity.

Further analysis by immunofluorescence microscopy showed minimal TNFR1 clustering in uninfected cells, increased clustering in cells infected with *H. pylori* WT or ∆PldA-in, and the most pronounced clustering in cells infected with ∆PldA (Figure 5e). Although the number of clusters formed did not differ significantly (Figure 5f), there was an increase in the size of clusters (Figure 5g) and intensity density (Figure 5h) of TNFR1 clusters in cells infected with ∆PldA, as compared to other groups.

We assessed how disrupting membrane integrity with methyl-β-cyclodextrin (MβCD) affects TNF-TNFR1 signaling.^41^ We demonstrated that cells treated with MβCD and infected with either WT, ΔPldA, or ΔPldA-in had significantly reduced activation of p38-MK2 (Fig. S7A). Additionally, p-RIP1 (S321) signals were barely detectable in MβCD-treated cells (Fig. S7A). These findings reinforce the importance of the integral membrane in regulating the activity of p38. An additional test using Dynole 34–2 revealed consistent p-p38 and p-MK2 signaling activation across conditions with or without Dynole 34–2, indicating endocytosis was not the major factor in TNFR1-mediated p38 signaling (Fig. S7B). Moreover, MβCD treatment significantly reduced TNFR1 clustering in cells infected with ΔPldA, resulting in levels close to those observed in cells infected with WT (Fig. S7C and S7D).

We investigated whether the presence of PldA, which disrupts membrane integrity and activates TNFR1, affects RIP1 ubiquitination recruitment.^42^ Using anti-RIP1 immunoprecipitation we showed that RIP1 ubiquitination was much lower in WT-infected cells than in ΔPldA-infected cells but was restored in ΔPldA-in infected cells (Figure 5I). MβCD treatment has also been shown to disrupt the ubiquitination of RIP1 and therefore affect the activation of p38 MAPK.^43^ The results together suggest that PldA and MβCD can disrupt TNFR1 clustering, weakening the p38-MK2 checkpoint response due to membrane disturbances through alteration of the lipid composition in the host macrophage.

### PldA-mediated p38-MK2-RIP1 signaling induces macrophage apoptosis

**Figure 6.**
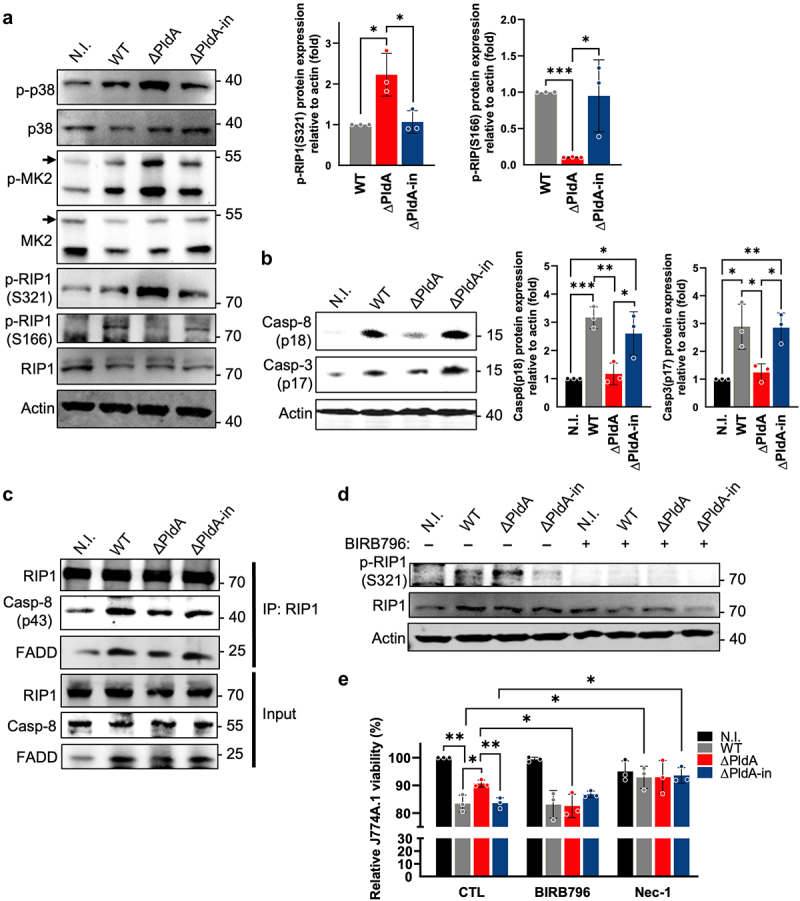
The presence of PldA inhibits macrophage p38-MK2 activation and induces RIP1-dependent apoptosis. (a to c) J744A.1 cells were infected with WT, Δ PldA, or ΔPldA-in at a MOI of 100 for 3 hr. (a) the levels of p38, p-p38, p-RIP1 (S321), p-RIP1 (S166), and RIP1 were detected by immunoblotting. Actin was used as an internal control. Quantification of ratio of p-RIP1 (S321) to actin and ratio of p-RIP1 (S166) to actin was displayed as the middle and right panel respectively. (b) the levels of active caspase-8 (p18) and caspase-3 (p17) were also detected by immunoblotting. Actin was used as an internal control. Quantification of ratio of active caspase-8 (p18) to actin and ratio of active caspase-3 (p17) to actin was displayed as the middle and right panel respectively. (c) Co-immunoprecipitation with anti-RIP1 from lysates was carried out to detect caspase-8 (p43) and FADD levels in RIP1-associated complex II. (d) J744A.1 was treated with or without BIRB-796 (100 nM) for 30 min, followed by infection with different *H. pylori* strains (WT, ∆PldA, or ∆PldA-in) at a MOI of 100 for 3 hr. The levels of p-RIPK1 (S321) and RIPK were determined by immunoblotting. Actin was used as an internal control. (e) J744A.1 was treated with DMSO (CTL), BIRB-796 (100 nM), or nec-1 (50 μM) for 30 min, followed by infection with various *H. pylori* strains (WT, ∆PldA, or ∆PldA-in) at a MOI of 100 for 3 hr. Assays using trypan blue exclusion were used to determine the number of live cells. Data presented are means ± SD from 3 independent experiments. Statistical significance was calculated using a two-tailed unpaired Student’s *t*-test. *p < .05, **p < .01, **p < .001.

We investigated whether PldA affects p38-MK2 signaling to regulate cell viability, as MK2 signaling contributes to cell fate.^44^ We found that infection with ΔPldA enhanced the p38 phosphorylation compared to infection with WT or ΔPldA-in (Figure 6a). Moreover, there was a stronger signal of p-RIP1 (S321) in ΔPldA-infected macrophages (Figure 6a). In contrast, there was little phosphorylation of p-RIP1 (S166) detected in macrophage infected with ΔPldA (Figure 6a). This is consistent with previous findings that p-RIP1 (S321) inhibits RIP1 autophosphorylation (p-RIP1 (S166)), which is a RIP1-dependent marker of cellular pathologies.^45,46^

After infecting cells with ΔPldA, we observed a significant decrease in active caspase-8 (p18) and caspase-3 (p17) when compared to both WT and ΔPldA-infected cells (Figure 6b). We then investigated if an apoptotic complex, which includes RIP1 and FADD,^45,47^ was involved. The results of immunoprecipitation experiments with anti-RIP1 from lysates of infected cells showed lower levels of caspase-8 (p43) and FADD in ΔPldA-infected cells than in those infected with WT or ΔPldA-in (Figure 6c), suggesting the involvement of RIP1-dependent apoptosis.

The phosphorylation signals of RIP1 at S321 were effectively eliminated when p38 was inhibited by BIRB796 (Figure 6d). Additionally, the use of BIRB796 resulted in a similar reduction in cell viability across all infected macrophages (WT, ΔPldA, and ΔPldA-in) (Figure 6e). Another p38 inhibitor, SB203580, also decreased the phosphorylation levels of RIP1-S321 (Fig. S8A). We tested the effects of Nec-1, a RIP1-specific inhibitor,^48^ on macrophage viability. Comparable levels were observed across all groups, including uninfected cells (Figure 6e). This was supported by decreased levels of p-RIP (S166) and active caspase-8 (p18) in the Nec-1 treated group (Fig. S8B and S8C). Our findings suggest that PldA primarily targets the p38-MK2-RIP1 pathway in macrophage membranes, causing RIP1-dependent apoptosis.

### PldA suppresses p38-MK2 activation in primary macrophage

**Figure 7.**
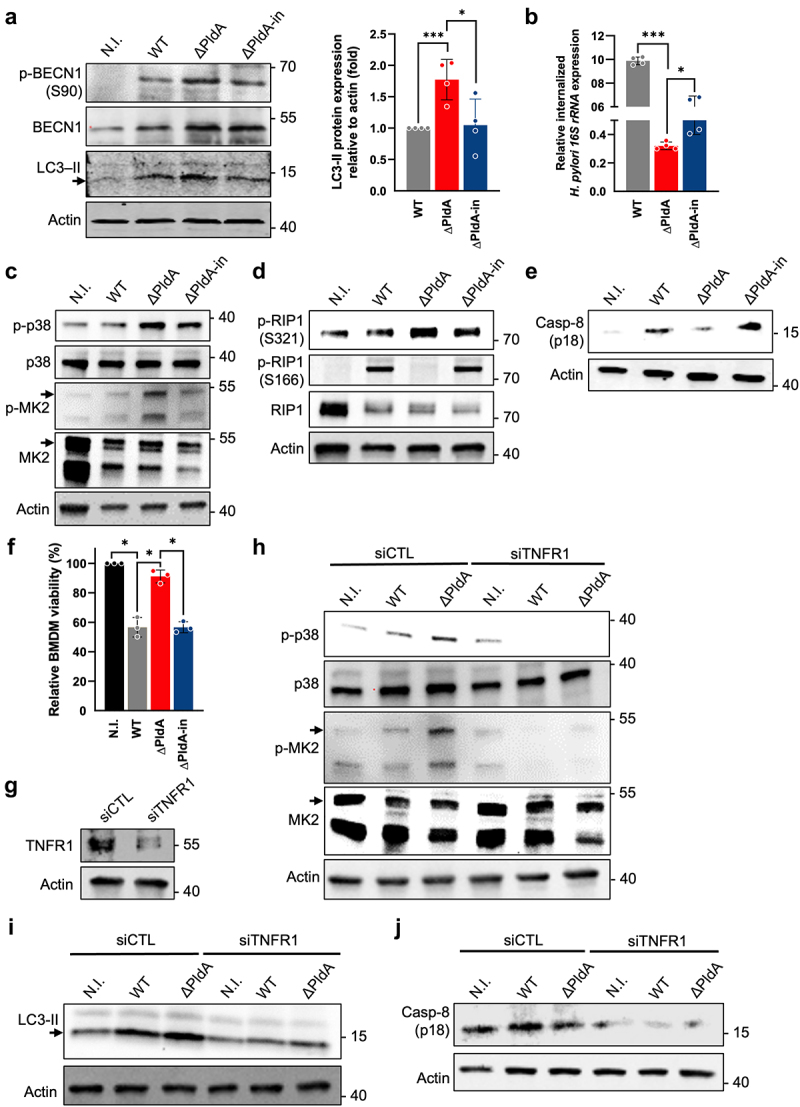
PldA inhibits p38-MK2 activation in primary macrophage. (a to e) BMDMs were either uninfected (N. I.) or infected with WT, Δ PldA, or ΔPldA-in at a MOI of 100 for 3 hr. The levels of LC3-II, p-BENC1 (S90), and BECN-1 were detected by immunoblotting (a, left panel). LC3-II to actin ratio is determined (a, right panel). A gentamycin bacterial *16s rRNA* assay was used to determine the internalization activity of *H. pylori* in BMDMs (b). Immunoblotting was used to detect the levels of p38, p-p38, MK2, and p-MK2 (c), the levels of p-RIP1 (S321), p-RIP1 (S166), and RIP1 (d), and the levels of active caspase-8 (p18) (e). Actin was used as an internal control. 3 independent experiments were conducted to obtain the data presented here. (f) trypan blue exclusion assays were used to determine the number of live cells in uninfected (N.I.) or infected with various *H. pylori* strains (WT, Δ PldA, or ΔPldA-in). (g) siTNFR1 transfected into BMDM and the expression of TNFR1 was verified using immunoblotting. Actin was used as an internal control. (h to j) siCTL and siTNFR1 BMDMs were either uninfected (N. I.) or infected with WT or Δ PldA at a MOI of 100 for 3 hr. Immunoblotting was used to detect the levels of p38, p-p38, p-MK2 and MK2 (h), the levels of LC3-II (i) and the levels of active caspase-8 (p18) (j). Actin was used as an internal control. Each value represents the mean ± SD from 3 independent experiments. Statistical significance (α = 0.05) was calculated using a two-tailed unpaired Student’s *t*-test. *p < .05, **p < .01, **p < .001.

A primary murine macrophage from bone marrow (BMDM) was used to validate the pathological effects of *H. pylori* PldA. We showed that BMDMs infected with WT or ΔPldA-in had lower levels of p-BECN1 (S90) than those infected with ΔPldA (Figure 7a). In addition, there were significantly lower signals of LC3-II in infection with WT or ΔPldA-in than those with ΔPldA infection (Figure 7a). The presence of PldA increased the level of bacterial *16s rRNA* detected in infection of WT or ΔPldA-in (Figure 7b), indicating a higher number of internalized bacteria. The levels of p-p38 and p-MK2 were lower in WT- or ΔPldA-in-infected BMDMs than in ΔPldA-infected BMDMs (Figure 7c). This was accompanied by a decrease in p-RIP1 (S321) and an increase in p-RIP1 (S166) in BMDMs infected with either WT or ΔPldA compared to ΔPldA (Figure 7d). Additionally, there was a higher level of the active caspase-8 (p18) in infection with WT or ΔPldA-in than with ΔPldA (Figure 7e). The cell viability assay revealed that PldA presence caused increased cell death in infected BMDMs (Figure 7f). Upon knocking down TNFR1, the infected BMDMs showed reduced autophagy indicated by the LC3-II level and attenuated p38 activation indicated by p-p38 levels (Figures 7g, i). Furthermore, the depletion of TNFR1 significantly reduced active caspase-8 (p18) levels, indicating less apoptosis (Figure 7j). The depletion of p38 using siRNA led to a substantial decrease in LC3-II levels (Fig. S9A and S9B) and an increase in active caspase-8 (p18) levels (Fig. S9C) when macrophages were infected with either WT or ΔPldA. Collectively, our findings indicate that PldA influences the TNFR1-p38-MK2 signaling pathway in infected BMDMs, which is consistent with our observations in J744A.1.

### *Suppression of PldA reduces bacterial survival and alters macrophage responses both in vitro and* in vivo

**Figure 8.**
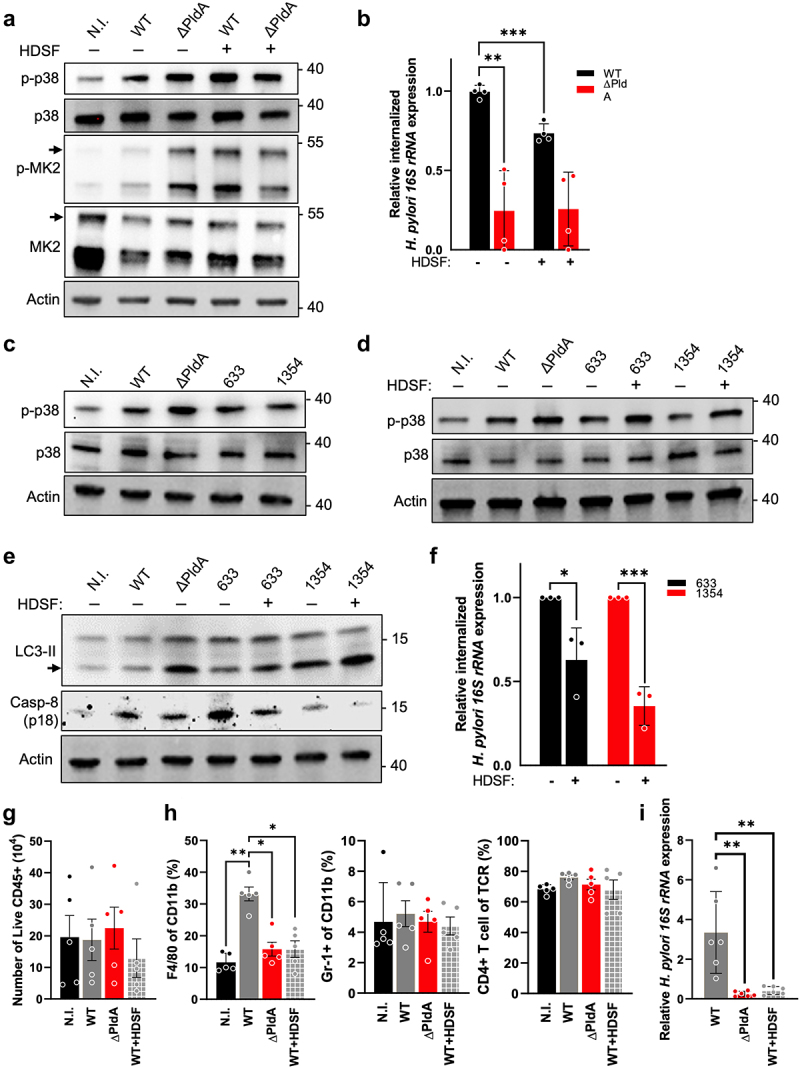
Suppression of PldA reduces bacterial survival and alters macrophage responses both in vitro and in vivo. (a to b) the *H. pylori* strains were treated with or without HDSF (1 mm) for 1 hr, followed by non-infection or infection with various strains of H. pylori (WT or ∆PldA) at a MOI of 100 for 3 hr. The levels of p-p38, p38, p-MK2 and MK2 levels were detected by immunoblotting analysis. Actin was used as the internal control (a). Infected cells were treated with gentamycin (100 μg/ml), and the level of *H. pylori* internalized in macrophages was determined using a quantitative PCR analysis (b). (c) J774A.1 cells were infected with different strains of *H. pylori* (WT, ∆PldA, v633 or v1354), and immunoblotting assays were conducted to determine the level of p-p38 or p38. Actin was used as the internal control. (d to e) *H. pylori* was pretreated with HDSF (1 mm) for 1 hr, followed by non-infection or infection with various *H. pylori* strains (WT, ∆PldA, v633 or v1354) at a MOI of 100 for 3 hr. Immunoblotting was used to detect the levels of p38, p-p38 (d), LC3-II, and active caspase-8 (p18) (e). Actin was used as an internal control. (f) J774A.1 cells were infected with different strains of *H. pylori* (v633 or v1354). The infected cells were treated with gentamycin (100 μg/ml), and levels of H. pylori internalized in macrophages were assessed using qPCR analysis. Each value represents the mean ± SD from 4 (b) or 3 independent experiments. 633, v633; and 1354, v1354. C57BL/6 mice received orogastric inoculations every 2 days with either WT *H.* *pylori*, ΔPldA, or WT *H. pylori* pre-treated with HDSF (1 × 10^9^ CFU), resulting in 4 total inoculations. (g) quantification of CD45+ leukocyte infiltration in gastric tissues following the regimen described. (h) infiltration levels of F4/80+/CD11b+ macrophages (left), Gr-1+/CD11b+ neutrophils (middle), and CD4+ T-cells (right) in gastric tissues following the regimen described. (i) gastric tissue bacterial loads of *H. pylori* assessed using qPCR, normalized to *gapdh*. (b and f) statistical significance (α = 0.05) was calculated using a two-tailed unpaired Student’s t-test. (g to i) data points represent individual mice, with bars indicating mean ± SEM collated from 3 independent experiments. Statistical analysis involved the Kruskal-Wallis test and subsequent Dunn’s post hoc test. *p < .05, **p < .01, ***p < .001.

We investigated reversing the response in WT-infected cells to mimic ΔPldA-infected cells by blocking PldA with the enzyme inhibitor, hexadecylsulfonyl fluoride (HDSF).^49,50^ We found that macrophages infected with HDSF-treated WT displayed similar activation of p38-MK2 signaling as those infected with ΔPldA (Figure 8a). We conducted a test on uninfected cells by treating them with HDSF, and the results indicated no significant changes in p38 phosphorylation levels when compared to the control group (Fig. S10A). This indicates that HDSF effectively inhibits PldA’s enzymatic activity in *H. pylori* without directly affecting p38-MK2 in macrophages. To further evaluate the effectiveness of HDSF in inhibiting PldA’s enzymatic activity, we conducted a time-course analysis to assess the CAG/CG ratio in *H. pylori* following HDSF treatment. The results showed that the CAG/CG ratio in HDSF-pretreated WT *H. pylori* remained comparable even after 3 hr of treatment (Figure S10B), confirming that HDSF maintains its inhibitory effect on PldA over time (Fig. S10B). We also tested whether HDSF treatment affects *H. pylori* viability by performing a CFU assay. The results showed that HDSF treatment did not significantly reduce bacterial viability (Fig. S10C), indicating that the inhibition of PldA activity by HDSF does not compromise the overall viability of *H. pylori*. To further characterize whether PldA’s enzymatic action affects macrophage response, we evaluated the effects of PldA-generated products CAG and LPE. When CAG and/or LPE were used, p38 signaling was minimally affected, indicating that PldA’s direct products are not the primary drivers of changes in p38 signaling during infection (Fig. S10D).

We next conducted an evaluation of the effect of HDSF on *H. pylori*'s intracellular survival. Treatment with HDSF decreased the intracellular survival of WT *H. pylori* in macrophages compared to the control (Figure 8b). There was no difference in survival for ΔPldA-infected macrophages, regardless of HDSF treatment. To investigate the potential of utilizing PldA inhibition as a therapeutic strategy against drug-resistant *H. pylori* infections, we evaluated the effect of HDSF on aberrant p38-MK2 signaling in macrophages infected with multi-drug resistant (MDR) clinical isolates v633 and v1354, which are derived from therapy-failed patients.^21^ We found that infections with either v633 or v1354 consistently decreased the phosphorylation levels of p38, similar to the effect observed in WT-infected cells (Figure 8c). Interestingly, when pre-exposed to HDSF, MDR strains v633 and v1354 exhibited increased levels of p-p38 (Figure 8d), increased levels of LC3-II, and decreased levels of active caspase-8 (p18) (Figure 8e) in macrophages infected with v633 or v135. Following HDSF treatment, there was a notable reduction in the intracellular survival of both v633 and v1354 in macrophages (Figure 8f).

Furthermore, we sought to understand the structural basis behind PldA’s interaction with HDSF, given our experimental results. PldA is commonly conserved in Gram-negative bacteria.^51,52^ Based on the structure of *E. coli* OMPLA,^53^ which shows a crucial dimeric active- state conformation, we used AlphaFold^54^ to create structural models of PldA that included different bacterial species such as *H. pylori*, *Campylobacter jejuni*, *Klebsiella pneumoniae*, and *Proteus vulgaris* (Fig. S10E). Our models revealed the presence of the HisXSer motif in the active site, which is where HDSF binds to the hydrophobic dimeric interface (Fig. S10F and S10G). The prediction analysis of *H. pylori* PldA reveals the His-X-Ser motif’s positioning on the outer surface of the bacterial membrane (Fig. S10G). This suggests that the motif is accessible for interaction with host macrophage membrane phospholipids, indicating a potential role of PldA in disrupting host membrane integrity and supporting *H. pylori* survival. Furthermore, the conservation of this motif across various bacterial species (Fig. S10H) indicates that PldA could represent a promising therapeutic target.

To extend our understanding of PldA’s role in immune modulation and bacterial virulence, we performed *in vivo* experiments where mice were infected with WT, ∆PldA, or WT *H. pylori* treated with the PldA inhibitor HDSF, following the same time course as shown in Figure 1a. We first examined the effect of PldA on *H. pylori* adhesion to gastric tissue. Our results revealed no significant difference in the adhesion capacity of the ∆PldA strains at 3 and 7 days post-infection (Dpi) (Fig. S10I). While total leukocyte counts were similar across groups (Figure 8g), infections with ∆PldA and HDSF-treated WT showed a marked decrease in macrophage numbers (Figure 8h). Neutrophil and CD4+ T-cell numbers in gastric tissues were consistent across all groups (Figure 8h). Moreover, *H. pylori* levels were elevated in WT-infected mice compared to those infected by ∆PldA or HDSF-treated WT *H. pylori* (Figure 8i). This was associated with an increased inflammatory response in WT-infected gastric tissues, as indicated by elevated levels of pro-inflammatory cytokines including *tnfα*, *il-6* and *il-1β* (Fig. S10J). These findings indicate that PldA enhances macrophage recruitment while simultaneously suppressing their ability to eliminate *H. pylori*. Our results together suggest the significant role of PldA in promoting bacterial persistence during infections. As PldA is conserved across Gram-negative bacteria, our study suggests that inhibiting it could help counter the effects of p38-MK2 signaling, thereby reducing the intracellular survival of drug-resistant strains. Additionally, it could provide insights into the underlying mechanisms of *H. pylori* pathogenesis and resistance.

### Discussion

Studies have shown that the enzyme PldA, found in Gram-negative bacteria, plays a vital role in their virulence. PldA is essential for the survival of many pathogens such as *Coxiella burnetii*, *Shigella flexneri*, and *Campylobacter* species.^55–57^ However, the effect of *H. pylori* PldA on the host’s immune system and its interference with macrophage signaling is not yet fully understood. This study reveals that *H. pylori* PldA leads to “membrane phospholipid perturbation,” which is a new molecular pathogenesis pattern that inhibits p38-MK2 activation in macrophages. PldA is a vital and conserved virulence factor that suppresses p38-MK2 signaling in two ways during *H. pylori* infection. Firstly, it reduces p38-MK2-mediated beclin 1 phosphorylation, which leads to the inhibition of autophagosome formation in macrophages, and thereby hinders pathogen clearance. This confirms *H. pylori*'s ability to impair autophagic flux, as previously reported by studies such as Zhang, 2018 and Lai, 2018.^30,58^ Secondly, PldA triggers the activation of RIP1 kinase, which induces macrophage death. In the absence of PldA or when p38 is pharmacologically inhibited, the phosphorylation of RIP 1 by MK2 is reduced. Therefore, PldA disrupts host autophagy and induces apoptosis via p38-MK2 signaling pathway.

Mechanistically, the presence of PldA markedly increases the production of LPE species and alters the LPE to PE ratio in macrophages. This suggests that PldA functions as an acyl hydrolase, specifically phospholipase in macrophages during infection. The elevated levels of LPEs potentially lead to membrane damage, thereby impacting the p38-MK2 signaling pathway in macrophages. Our findings support the concept that changes in membrane fluidity, influenced by PldA’s phospholipase activity, interfere with the ubiquitination of RIP1, particularly within the TNFR1 signaling pathway.

**Figure 9.**
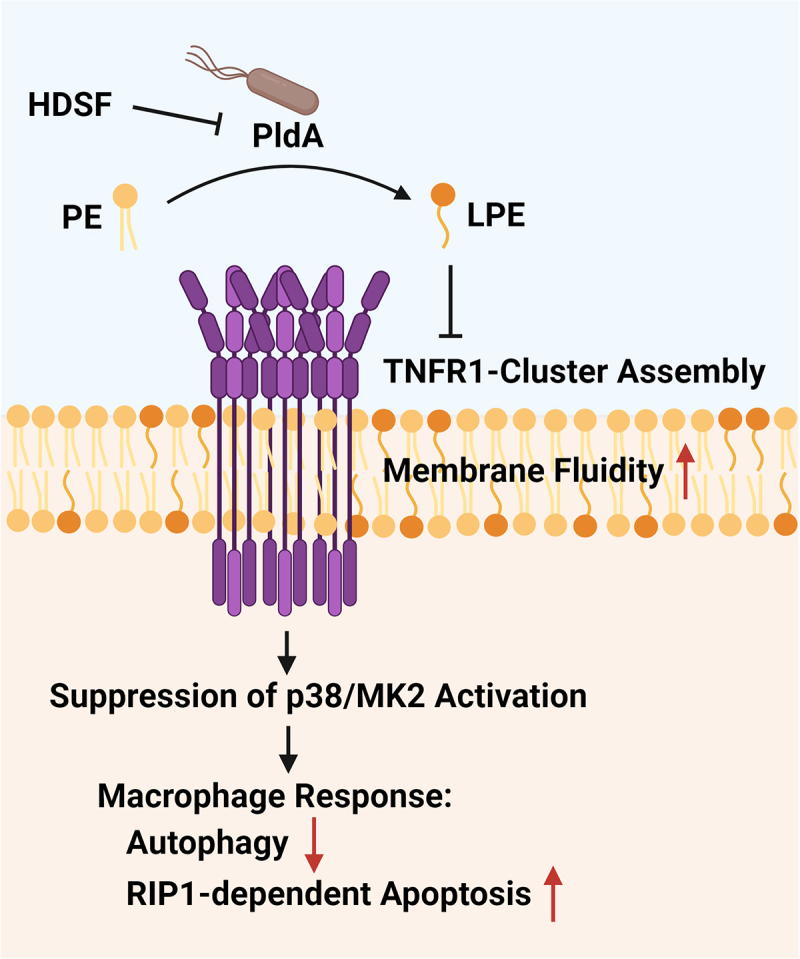
A proposed model of PldA-mediated dysregulation in macrophage defense against *H. pylori* infection. During *H. pylori* infection, the PldA enzyme is activated, which triggers the production of lysophospholipids. These lipids can damage the macrophage membrane, preventing the clustering of TNFR1, which is important for effective TNF-TNFR1 signaling. This disruption weakens the host’s immune defense, creating a favorable environment for the persistence of *H. pylori*.

The dynamic nature of plasma membranes, as depicted by the fluid mosaic model, is crucial in cell signaling. Variations in phospholipid composition, saturation levels, and the presence of lipid microdomains like lipid rafts significantly influence cellular signaling processes.^36,59^ For instance, disturbances in lipid rafts have been shown to play a vital role in TLR4 activation and overall immune signaling.^60^ Moreover, a recent study shows that a homoserine lactone, a *Pseudomonas aeruginosa* quorum-sensing metabolite, inserts into the plasma membrane, dissolves lipid domains, and triggers significant cell death by causing spontaneous TNFR1 trimerization.^61^ Our findings indicate that the TNFR1 antagonist inhibits p38 activation in macrophages infected with *H. pylori*, in line with the differential effects of TNFR1-p38-MK2-RIP1 signaling observed in infections with different PldA statuses. We also demonstrate that MβCD treatment that disrupts raft structure diminishes p38-MK2 activation. These results support the hypothesis proposed by Morton et al. that cholesterol presence and an organized bilayer membrane structure are critical for enabling TNF-TNFR1 clusters to undergo necessary conformational changes, thus initiating downstream signaling in macrophages.^41^ Furthermore, we found that infection with WT or PldA-in *H. pylori* disrupts membrane fluidity, which prevents the ubiquitination of RIP1. This alteration is particularly significant in the context of the TNFR1 pathway. Our observations support previous studies that highlighted the importance of cholesterol and structured membrane bilayers in modulating TNF-TNFR1 clustering and downstream signaling in macrophages.^41^ The complex interaction between *H. pylori* PldA and macrophage responses provides valuable insights into the mechanisms underlying phospholipid metabolism and its impact on host-pathogen interactions. The breakdown of membrane phospholipids by *H. pylori* PldA reduces membrane rigidity, preventing TNFR1 clustering from triggering downstream signaling pathways (Figure 9).

PldA is a protein that resides on the outer membrane of numerous Gram-negative bacteria.^26^ It has a crucial role in regulating the phospholipid turnover activity. The structure of PldA reveals that the catalytic and substrate-binding pockets are present in the dimeric interface of homodimers, but not in monomers.^53^ Its activity is controlled by dimerization and is calcium-dependent.^26^ Upon being activated by events such as heat shock, phage-induced lysis, small antimicrobial peptides, and EDTA treatment, PldA switches from an inactive monomer to an active dimer. This enables the binding of substrate and calcium in the active site, leading to the hydrolysis of phospholipids and the production of lysophospholipids and fatty acids. This process damages the outer membrane, making it easier for colicin,^62^ enterochelin,^63^ or fatty acids^64^ to be transported. Here, we show that the presence of PldA in an *H. pylori* infection can result in a significant increase in the production of LPE species and the ratio of LPE to PE in macrophages. We have also observed that both WT-infected macrophages and ΔPldA infections showed a considerable decrease in the CAG(14:0) to CG ratio. These findings indicate that PldA primarily exerts its phospholipase activity on macrophages during infection. The increased level of LPE can damage the membrane and affect the p38-MK2 signaling pathway associated with membrane deformation in macrophages. In addition, our microarray western analysis has also revealed that PldA can affect various signaling pathways, such as GSK3, PDK1, and cJUN. We have mainly focused on the p38 MAPK signaling pathway, which has been shown to regulate both autophagy and apoptosis.^37^ Our data suggests that *H. pylori* PldA regulates the p38-MK2 signaling pathway by breaking down membrane phospholipids into LPEs. PldA increases the levels of LPEs within macrophages by cleaving membrane phospholipids, leading to membrane damage that alters cellular signaling.

It is interesting that PldA displays a unique combination of acyltransferase and phospholipase functions, facilitating the pathogenicity of *H. pylori*. PldA, in concert with CGT, forms a strategic pair in cholesterol metabolism. They work together in a synergistic yet distinct manner to promote *H. pylori*'s immune evasion and host interaction. *H. pylori* targets cholesterol-rich microdomains in host cells, employing CGT to extract and glycosylate cholesterol.^12,15,65^ This activity not only aids in immune evasion but also induces a reorganization of the host membrane.^16,66,67^ Such reorganization is vital for the efficient delivery of virulence factors like CagA into epithelial cells, playing a significant role in manipulating host-pathogen interactions.^16^

PldA complements CGT’s function by acylating the CGs formed by CGT, resulting in acylated forms that significantly enhance *H. pylori*'s adherence to epithelial cells.^17^ This enhanced adhesion is an important factor in *H. pylori*'s ability to colonize the gastric mucosa effectively.^24^ Beyond its acyltransferase role, PldA operates as a phospholipase within macrophages as seen in this study, processing host phospholipids to produce lysophospholipids, predominantly LPEs. This dual enzymatic activity of PldA highlights its versatility and critical importance as a virulence factor, supporting *H. pylori*'s survival and persistent infection within the host. The synergy between PldA and CGT highlights the complex nature of *H. pylori*'s mechanisms for immune evasion and host interaction.

The issue of antimicrobial resistance is a global challenge, and it is crucial to understand its mechanisms.^3^ The PldA gene is prevalent in clinical isolates of MDR *Pseudomonas aeruginosa* and is linked to phenotypic resistance in *Campylobacter* spp.^68,69^ This illustrates its wider relevance. The role of PldA goes beyond *H. pylori* and impacts the pathogenesis of other Gram-negative bacteria by altering the composition of membrane lipids and influencing host immune responses. Our study showed that with a PldA inhibitor, reducing the intracellular survival of MDR *H. pylori* strains isolated from therapy-failure patients and macrophage apoptosis is possible. This suggests that PldA could be a potential target in combating *H. pylori*-associated diseases and resistance. Further investigation is necessary to fully understand PldA’s role in *H. pylori* pathogenesis and its potential impact on other Gram-negative bacteria, potentially regulating other immune responses.

## Materials and methods

### Cell line and bacteria strains

J774A.1 macrophage cells (ATCC TIB67) were cultured in Dulbecco’s Modified Eagle’s Medium (DMEM), supplemented with 10% heat-inactivated fetal bovine serum (HyClone, Logan, UT) and 5% CO_2_.

*H. pylori* 26695 (ATCC 700,392) was used as the reference strain in this study. *H. pylori* was routinely cultured on Brucella agar plates with 2.8% Brucella powder (Becton Dickinson, Franklin Lakes, NJ, USA), 1.5% agar (Cleveland, OH, USA), 0.2% β-cyclodextrin (Sigma-Aldrich, St. Louis, Missouri, USA), 0.1% yeast extract, 10% sheep blood, and 1% isovitalex (Becton Dickinson, Franklin Lakes, NJ, USA) in a microaerophilic atmosphere (5% O_2_, 10% CO_2_, and 85% N_2_) at 37°C for 2 days. Different isogenic *H. pylori* mutant knockout strains (ΔPldA, ΔCGT, ΔVacA, ΔCagA) and a PldA-complementation strain (ΔPldA-in) was utilized in this study.

### BMDM differentiation and treatment

Mice were maintained in a pathogen-free colony with a 12-hr cycle of light and dark (7 a.m. to 7 p.m.), and fed chow ad libitum. The C57BL/6 animals were purchased from National Laboratory Animal Center. Mice were grown to an age of 6 to 8 weeks before being sacrificed. The National Tsing Hua University IACUC approved (approval number: NTHU-IACUC-110075) all animal experiments in accordance with the regulatory standards.

Femur bones were used to harvest the bone marrow cells. Cell suspensions were filtered through a 70 mm nylon strainer into a 50 mL tube. A 5 mL solution of ACK lysis buffer (Thermo Fisher) was added to lyse the red blood cells for 2 min at room temperature. Following lysis, 20 mL of RPMI with 2% FBS was added, and the tube was centrifuged at 100 rcf for 5 min at 4°C. The pellet was washed and resuspended in BMDM medium (10% heat-inactivated FBS, 10 ng/ml recombinant murine M-CSF (Biolegend, cat#576402) in DMEM medium, with 1% penicillin/streptomycin). The cells were then plated at 2.5‒3×10^6^ cells per well in a sterile 6-well tissue culture plate and cultured for 7 days. BMDMs were collected on day 7 of differentiation and plated for experiments in DMEM containing 10% FBS.

### Mice infection and tissue harvest

C57BL/6 mice, aged 6 to 8 weeks, were utilized for all *in vivo* studies. Prior to inoculation, *H. pylori* colonies were harvested to achieve a concentration of 1 × 10^9^ CFU and resuspended in PBS. Mice were subsequently orogastrically inoculated with the *H. pylori* suspension every other day, totaling 4 inoculations. Upon sacrifice, various tissues were harvested for subsequent analyses. The stomach was excised between the esophagus and the duodenum, laid open, and gently rinsed with cold PBS. Each stomach sample was then partitioned for flow cytometry and bacterial clearance assays. For RNA isolation, gastric tissues were preserved in TRIzol solution. Tissue DNA extraction was performed using the Nucleospin Tissue Mini kit (Macherey-Nagel 740,952.50) following the manufacturer’s protocol. The detection of *H. pylori* load in gastric tissue and the internal control *18s rRNA* was conducted using the primers listed in Table S4.

### Isolation of immune cells from gastric tissue

After sacrificing the mice, gastric tissue was excised and thoroughly rinsed with PBS. The cleaned tissue was then incubated in HBSS supplemented with EDTA, undergoing two successive rounds of incubation, each lasting between 30 to 40 min. Following this, the tissue was resuspended in a solution containing collagenases and DNase to aid digestion. Concurrently, cells from the lymph nodes were isolated by gently pressing the tissue through a 70 μm strainer. Finally, to eliminate erythrocytes, cells obtained from both the gastric tissue and lymph nodes were treated with ACK lysis buffer.

### Immune cell phenotyping and flow cytometry analysis

Tissue samples were first treated with a blocking buffer that contained the anti-CD16/CD32 Fcγ receptor blocking antibody. Subsequently, cells were stained using a combination of antibodies directed against various mouse surface markers. For intracellular staining procedures, cells were initially fixed with paraformaldehyde. After fixation, they were permeabilized using a permeabilization buffer, which was followed by incubation with intracellular antibodies. Flow cytometry analysis was conducted using the Cytek® Aurora/Northern Lights system, and the resultant data were analyzed utilizing the SpectroFlo software.

### Antibodies and reagents

The antibodies and reagents used in this study are described in Table S2 and Table S3 respectively.

### Immunoblotting and immunoprecipitation

The lysates were prepared by lysing the cells in RIPA buffer (20 mM TrisHCl pH 7.4, 150 mM NaCl, 1 mM EDTA, 1% Triton X-100, 10% glycerol, 0.1% SDS, and 0.5% deoxycholate) supplemented with complete protease inhibitor (Santa Cruz) and phosphatase inhibitor, PhosSTOP (Sigma). After this, the cell lysates were mixed with an appropriate amount of sodium dodecyl sulfate-polyacrylamide gel electrophoresis (SDS PAGE) loading buffer (100 mM SDS Tris (pH 6.8) 4%, bromophenol blue, 0.2% glycerol, 20% 2-mercaptoethanol) and incubated at 100°C for 10 min.

SDS-PAGE was run using an equivalent amount of protein. The proteins were transferred to PVDF membranes (Millipore) and blocked with 3% BSA in Tris-buffered saline. The membranes were then incubated with the primary antibody in 3% (wt/vol) BSA in 0.1% Tween-20 in Tris-buffered saline and then with HRP-conjugated secondary antibodies in the same solution. The blots were developed using Trident Femto Western HRP substrate (Genetex) and captured with iBright FL1500 Imaging System (Thermo Fisher Scientific).

Immunoprecipitation was performed by lysing cell pellets in IP lysis buffer (50 mM Tris-HCl (pH 7.4), 150 mM NaCl, 0.5% NP40, and protease inhibitor) at 4°C for 1.5 hr. An appropriate amount of cell lysates, the respective antibodies (1 μg), and 10 μl of PureProteome protein A/G magnetic beads (Millipore) were incubated and gently shaken overnight at 4°C. The beads were then washed 3 times in IP wash buffer (137 mM NaCl, 2.7 mM KCl, 10 mM Na_2_HPO4, 1.8 mM K_2_HPO_4_, 0.1% Tween 20, pH 7.4). The complexes were then eluted with IP lysis buffer at 85°C for 10 min. After SDS-PAGE fractionation, proteins were detected using immunoblotting.

### Measurement of LC3-II flux

Cells were seeded and incubated overnight. Before infection, CQ was added to the cells at a concentration of 10 μM. The cells were then infected with different strains of *H. pylori* for 3 hr before being harvested for subsequent immunoblotting analysis.

### Preparation of *H.* *pylori* lysate

*H. pylori* lysates were prepared using freshly cultured bacteria (1 × 10^8^), resuspended in DMEM cell culture medium, and lysed using sonication for 5 cycles of 15 s on, 15 s off, at 20% power (Misonix, Sonicator 3000) at 4°C. The samples were sonicated, and centrifuged, and the supernatants were collected.

### Quantitative PCR (qPCR) analysis of mRNA

The total RNA was extracted using TRizol reagent (Thermo Fisher Scientific). The cDNAs were prepared using the SuperScript III Reverse Transcriptase (Thermo Fisher Scientific), dNTP (Genedirex, Las Vegas, Nevada, USA), and random primers (Thermo Fisher Scientific). cDNA samples were detected using SensiMixTM SYBR® Hi-ROX Kit (Bioline, Taunton, MA, USA) and ABI StepOnePlus Real-Time PCR System (Thermo Fisher Scientific). The *gapdh* gene served as an internal control. The sequences of the primers are listed in Table S4.

### Micro-western array

A signaling pathway analysis was performed by the Micro-western Core facility of the NHRI, using appropriate antibodies as described previously.^70^

### RNA interference and gene transduction

The transfection of siRNA was performed using Lipofectamine RNAiMAX (Thermo Fisher Scientific 13,778,075) according to the manufacturer’s protocol. The commercial sources of siRNA are purchased from Dharmacon.

### Immunofluorescence and image analysis

J774A.1 cells (2 × 10^5^) were seeded in 6-well plates at 37°C for 16 hr. The cells were infected with WT, ΔPldA, or ΔPldA-in *H. pylori* at a MOI of 100 for 3 hr and fixed with 3.7% paraformaldehyde for 30 min. The cells were then permeabilized with 0.1% Triton X-100 for 30 min and then blocked with PBS containing 2.5% bovine serum albumin. Using specific probes or antibodies, nuclei, autophagosomes, and *H. pylori* were visualized.^71^ The cells were washed 3 times with PBS for 10 min, and then incubated at room temperature for 1 hr with corresponding secondary antibodies conjugated with fluorescent dye. Following 3 rinses with PBS for 10 min, the cells were mounted onto glass slides using mounting media. Following staining, cells were analyzed using a confocal laser scanning microscope (LSM 780, Carl Zeiss, Göttingen, Germany) with a 40× objective described previously^72^ processed with the Zen Blue software (Carl Zeiss). The quantification of puncta and *H. pylori* colocalization was carried out using Zen Black software (Carl Zeiss). The formation of LC3 puncta was calculated using the GFP-LC3 plugin in ImageJ, as described in.^73^ Cluster analysis of TNFR1 was measured using “Analyze Particle” plugin in ImageJ upon applying the appropriate threshold.

### Untargeted lipidomic profiling with liquid chromatography coupled with Q-TOF mass spectrometry

Hydrophobic metabolites were extracted using a modified MTBE method.^74^ Following this, the metabolites were redissolved in 500 μl of an IPA/acetonitrile/water (2:1:1, v/v/v) mixture. For untargeted metabolomic analysis, hydrophobic metabolites were analyzed using Waters ACQUITY BEH C18 column (1.7 μm × 2.1 mm × 100 mm) with temperature maintained at 60°C. In order to optimize the parameters, the mobile phase A consisted of acetonitrile/water (40:60, v/v) containing 10 mM ammonium formate, and the mobile phase B consisted of isopropanol/acetonitrile (90:10, v/v) containing 10 mM ammonium formate. The mass spectrometry was performed on a Waters Q-TOF-MS (SYNAPT G2S HDMS, Waters MS Technologies, Manchester, UK) that operated in the positive ion mode. MS data, such as retention times, m/z, and ion intensities, were analyzed using Progenesis QI (Waters Corp., Milford, MA). A comparison of our results with in-house data (standards based on retention times and mass spectra) was conducted to identify metabolites. Analysis of MS/MS data as well as comparison of spectral data with reference compounds confirmed the identity of the metabolites. The data were uploaded to the MetaboAnalyst® platform for clustering analysis using Pearson’s and Ward’s methods.

### Analysis of quantitative phospholipid profiles using liquid chromatography coupled with tandem mass spectrometry

Hydrophobic metabolites were redissolved in 500 μl of IPA/acetonitrile/water (2:1:1, v/v/v) containing an internal standard (330709W, Avanti Polar Lipids, Alabaster, AL, USA). Analyses were conducted using Waters ultra-high-performance liquid chromatography coupled with Waters Xevo TQXS mass spectrometry (Waters Corp.). Chromatographic separation conditions were the same as those used for untargeted lipidomic profiling. The capillary voltage was set to 1000 V for ESI positive ion mode and 500 V for ESI negative ion mode. The sampling cone voltage was set to 30 V. The desolvation gas flow rate was maintained at 1000 L/h, with the cone gas flow set at 150 L/h. The desolvation temperature was set to 500°C, and the source temperature was maintained at 150°C. Mass analysis was conducted using the Waters Xevo TQ-XS system in positive-ion ESI mode, with the mass transitions for analysis listed in Table S5. Data was acquired and analyzed by MassLynx and TargetLynx (Waters Corporation).

### Cell viability assay

Cells were seeded in a 6-cm plate and incubated overnight at 37°C for cell counting. Different bacteria strains were then used to infect the cells. Trypan Blue Dye was used to distinguish between live and dead cells. The number of live cells was then determined using Countess II FL Automated Cell Counter (Thermo Fisher Scientific).

### Bacterial gentamycin, adherence, and clearance assays

The gentamicin assay was conducted based on the methodology described by Lai et al.^30^ J774A.1 cells (1 × 10^5^) were seeded in 6-well plates and allowed to incubate for 16 hr at 37°C. Following this, cells were infected with WT, ΔPldA, or ΔPldA-in *H. pylori* strains using a multiplicity of infection (MOI) of 100 for 3 hr. Gentamycin was then introduced to each well for an additional 1.5-hr incubation. Following this, cells were rinsed with PBS and lysed using sterilized water. These lysates were then diluted and spread onto sheep blood agar plates. After an incubation period of 3‒5 days, *H. pylori* colonies were calculated. Results were presented in CFUs. For bacterial clearance assays, *H. pylori*'s total bacterial content was ascertained using its 16s ribosomal RNA (*16s rRNA*). The adherence assay involved washing the infected cells with warm PBS with mild agitation thrice. Following this, samples were collected to quantify bacterial presence using *H. pylori 16s rRNA*.

### Membrane lipid order and general polarization analysis

The membrane lipid order imaging of macrophage cells was performed as described previously.^40^ Briefly, cells were stained with 5 μM Laurdan (Cayman Chemicals, Cat. 19706) for 30 min at 37°C in a humidified incubator with 5% CO_2._ Following 3 washings in PBS, the cells were imaged using a confocal laser scanning microscope (LSM 780, Carl Zeiss, Göttingen, Germany) (excitation at 356 nm; emission at 400–460 nm and 470–530 nm). General polarization (GP) values were calculated by the formula: $P=\frac{I_{440-I_{490}}}{I_{440+I_{490}}}$. Pseudocolored GP images and HSB images were created using an ImageJ plug-in.^40^ HSB images that combined mean intensity and rainbow RGB color GP images were shown. ImageJ was used to quantify the GP values of cells from GP images. The frequency of pixels with each GP value was plotted as a histogram using GraphPad Prism 7.

### Thin layer chromatography

The extraction of cholesterol derivatives from *H. pylori* was accomplished essentially in the same manner as previously reported.^16^ Briefly, the bacteria were harvested by centrifugation (4°C, 10 min, 3200×g), followed by boiling in a water bath for 10 min. A 2:1 (v/v) chloroform/methanol solution was used to extract glycolipids, which were then washed by Folch partitioning. The organic phase was then evaporated. The residue was re-dissolved in ethyl acetate and subjected to Silica Gel-60 F254 TLC using a chloroform/methanol 85:15 (v/v) solvent system. Cholesterol and CGs were visualized by soaking in 15% sulfuric acid containing 10 mM n-naphthol and then heating to 160°C.

### Cibersortx Analysis

Microarray data (Accession Number: E-MTAB-8889) were used to analyze mRNA expression profiles of patients diagnosed with or without *H. pylori*.^28^ The expression profiles were analyzed using Cibersortx with the default settings. The immune populations of each patient were generated and analyzed using Graphpad Prism.

### Statistical analysis

The statistical analysis was conducted using GraphPad Prism 7 (GraphPad Software Inc., San Diego, CA). For *in vivo* infection studies, results represent the mean ± SEM and each point represents one mouse. The Mann-Whitney test was used to compare two groups, and the Kruskal-Wallis test was followed by Dunn’s post hoc test for comparisons between multiple groups as indicated. Results are presented as means ± SD, and the number of samples for each group is indicated in the figure legend. Unpaired Student’s *t*-test was used for all *in vitro* data analysis. Values of *p* < 0.05 were considered statistically significant.

## Abbreviations

PldA: phospholipase A
CG: cholesteryl-α-D-glycopyranoside
CGT: cholesterol-α-glycosyltransferase
VacA: vacuolating toxin A
CAG: cholesteryl-6-O-tetradecanoyl-α-D-glucopyranoside
TNF: tumor necrosis factor
TNFR1: tumor necrosis factor receptor 1
PC: phosphatidylcholine
PE: phosphatidylethanolamine
LPE: lysophosphatidylethanolamine
LC3: Microtubule-associated proteins 1 light chain 3B
CQ: chloroquine
WT: wild type
LPC: lysophosphatidylcholine
RIP1: Receptor-interacting serine/threonine-protein kinase 1
TLR: Toll-like receptor
MβCD: methyl-β-cyclodextrin
FADD: Fas Associated Via Death Domain
BMDM: bone marrow-derived macrophage
HDSF: hexadecylsulfonyl fluoride
MDR: Multiple drug resistance

## Supplementary Material

Supplemental Material

## Data and materials availability

All data are in the main text and/or the Supplementary materials. Metabolomics data are available upon request.
