# Supplementary figures and images for: Helicobacter pylori PldA modulates TNFR1-mediated p38 signaling pathways to regulate macrophage responses for its survival
Source: Gut Microbes. 2024 Oct 6;16(1):2409924. doi: 10.1080/19490976.2024.2409924 (PMC11457642; doi:10.1080/19490976.2024.2409924)

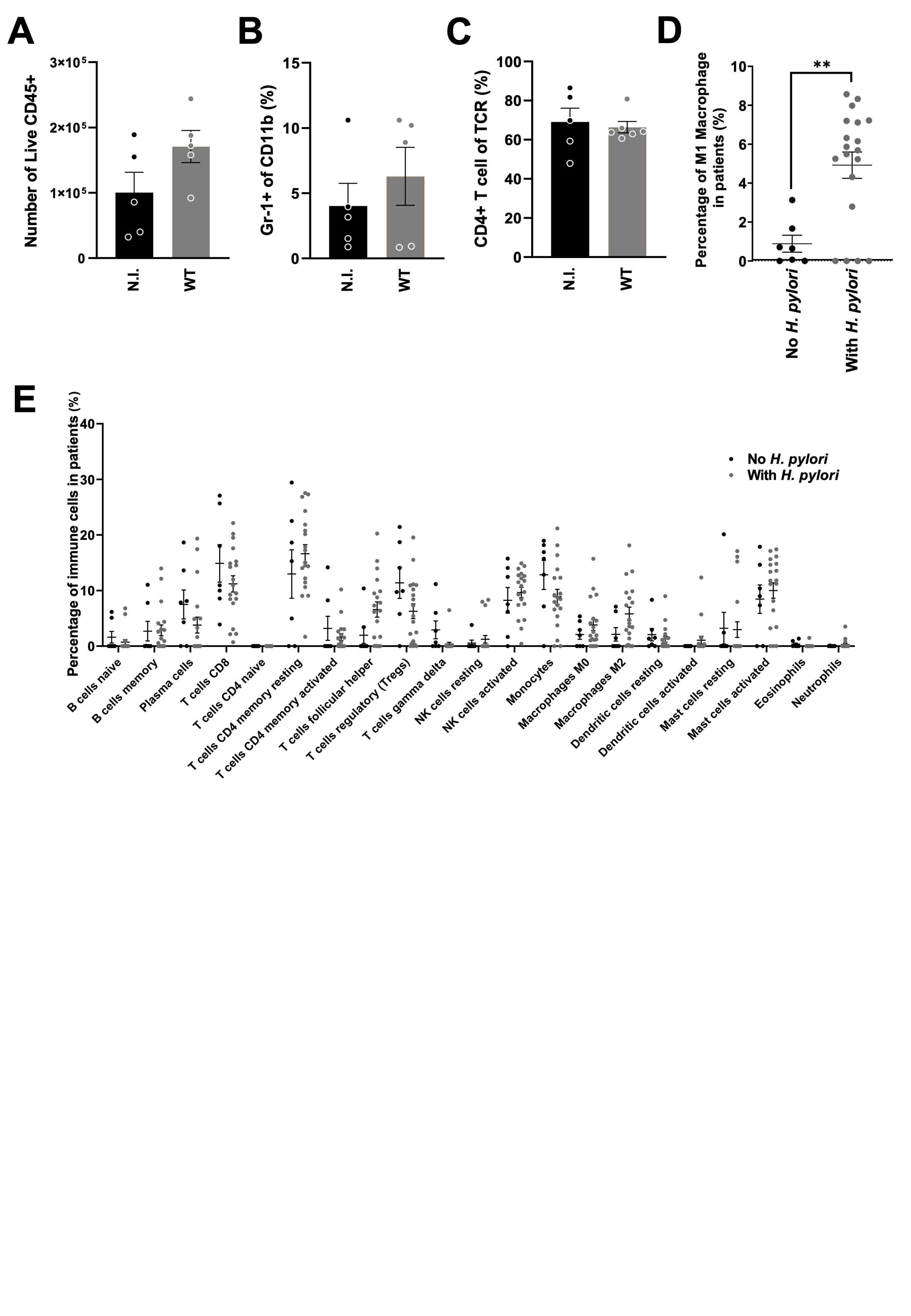

Supplement: Supplemental Material [file KGMI_A_2409924_SM9224.zip › High Res. Figures/High Res. Figures/Figure S1.tiff]

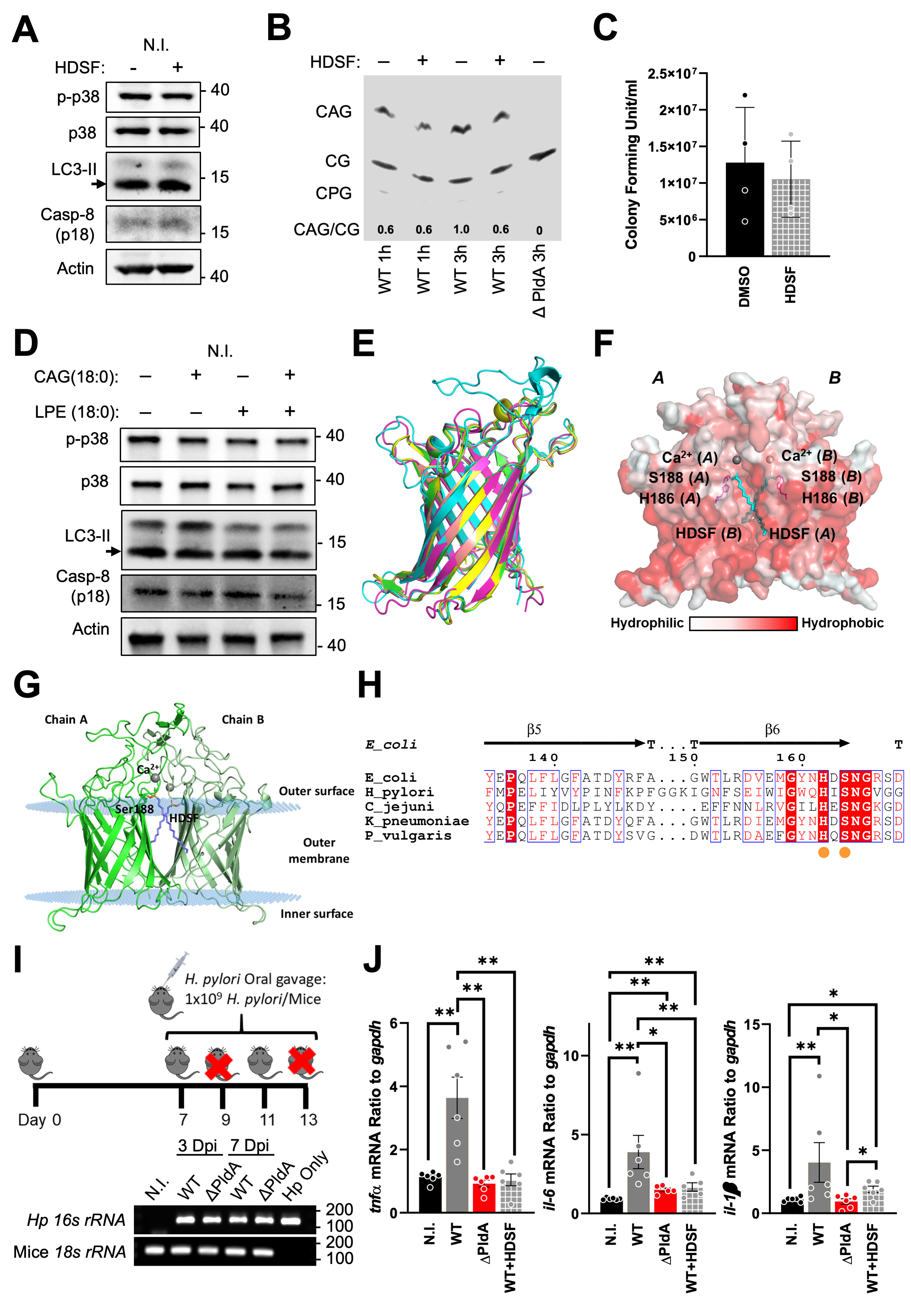

Supplement: Supplemental Material [file KGMI_A_2409924_SM9224.zip › High Res. Figures/High Res. Figures/Figure S10.tiff]

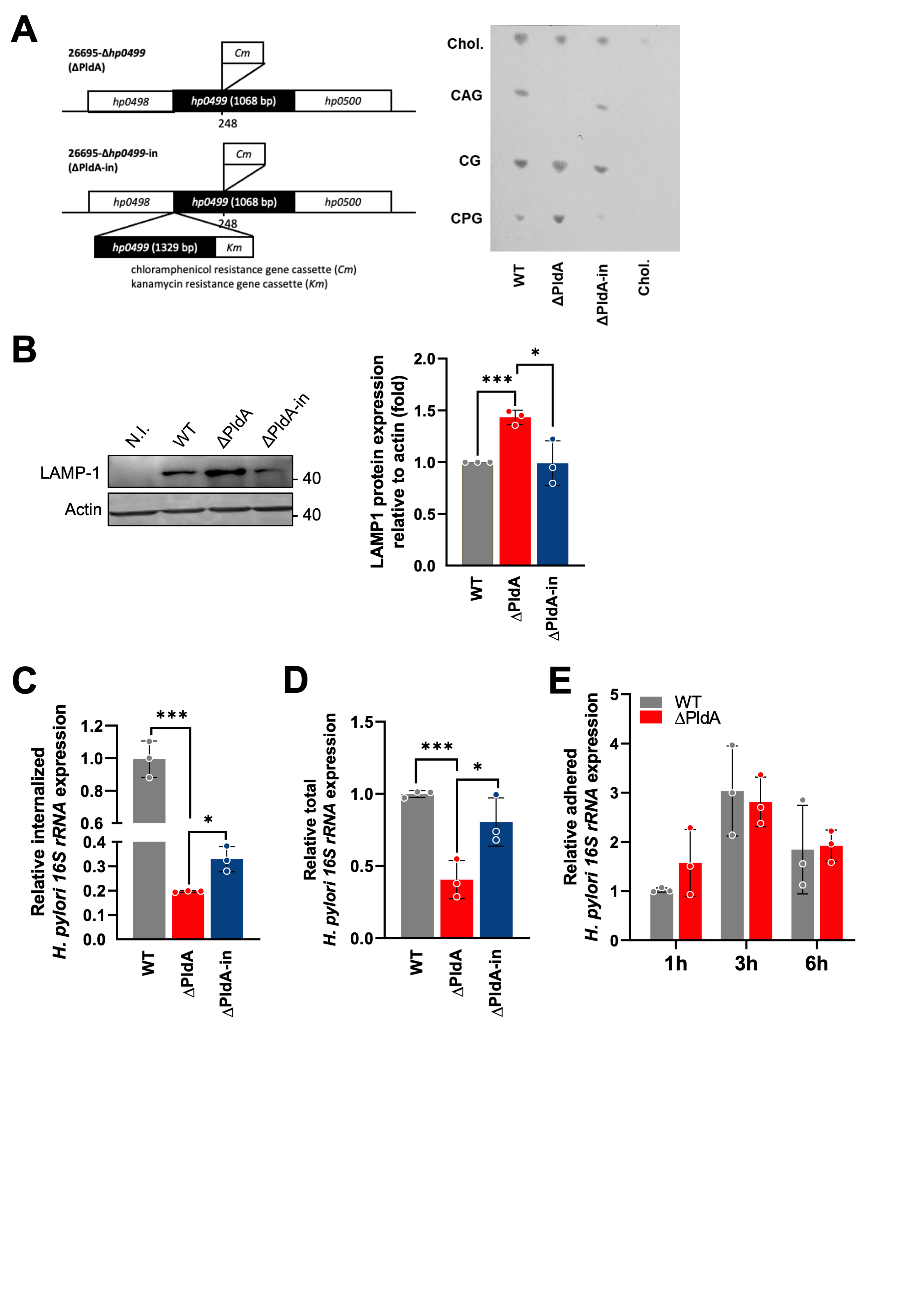

Supplement: Supplemental Material [file KGMI_A_2409924_SM9224.zip › High Res. Figures/High Res. Figures/Figure S2.tiff]

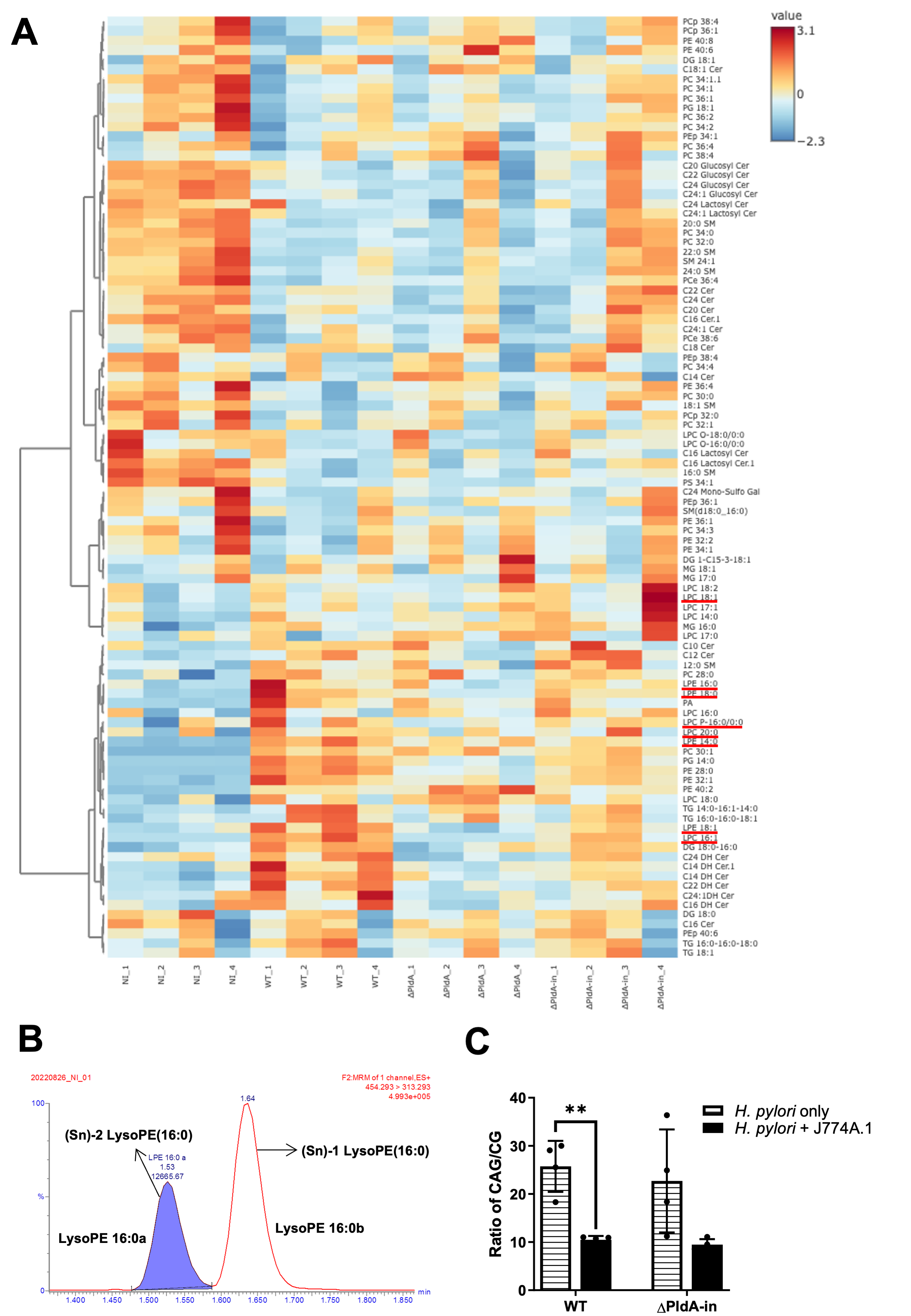

Supplement: Supplemental Material [file KGMI_A_2409924_SM9224.zip › High Res. Figures/High Res. Figures/Figure S3.tiff]

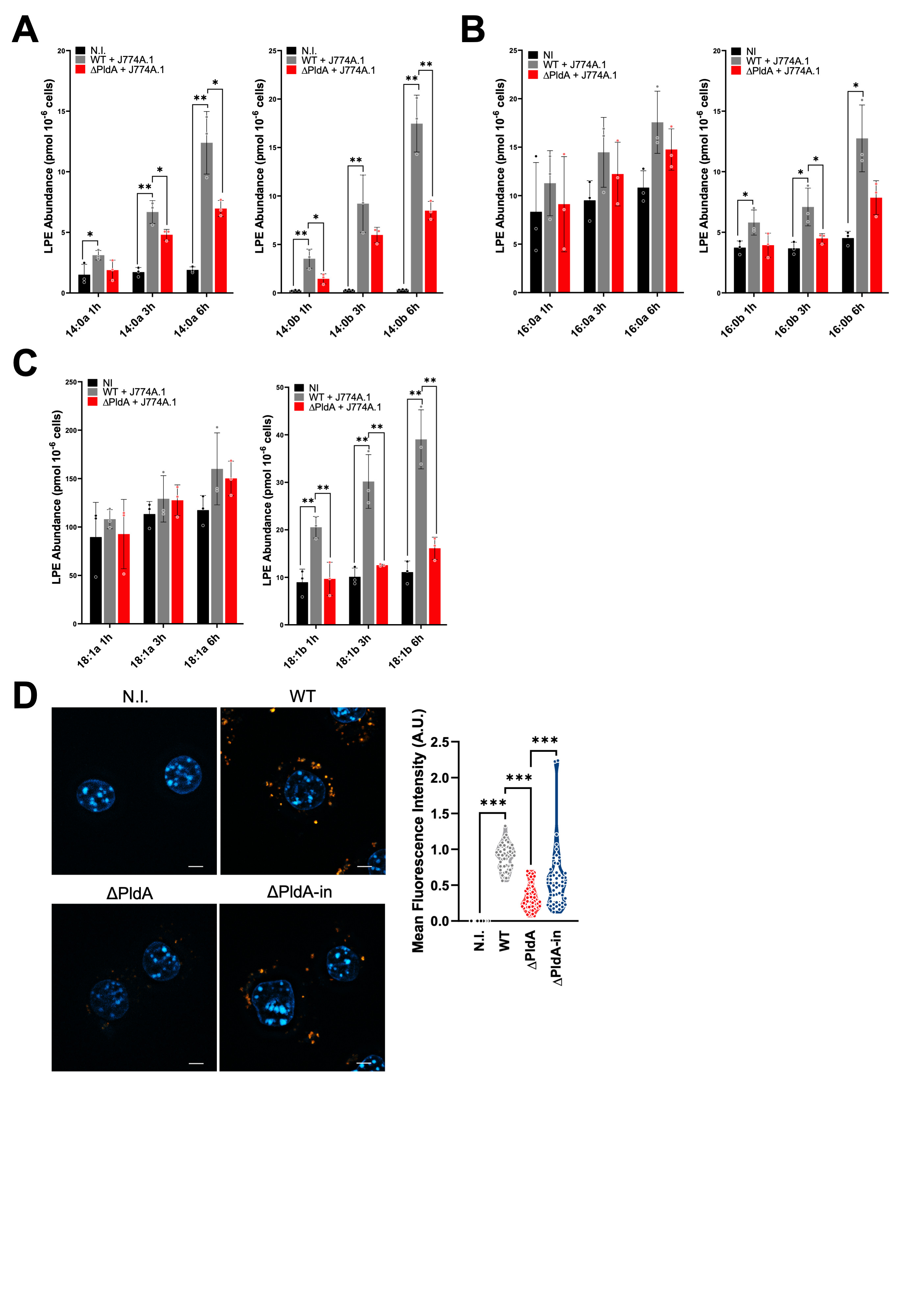

Supplement: Supplemental Material [file KGMI_A_2409924_SM9224.zip › High Res. Figures/High Res. Figures/Figure S4.tiff]

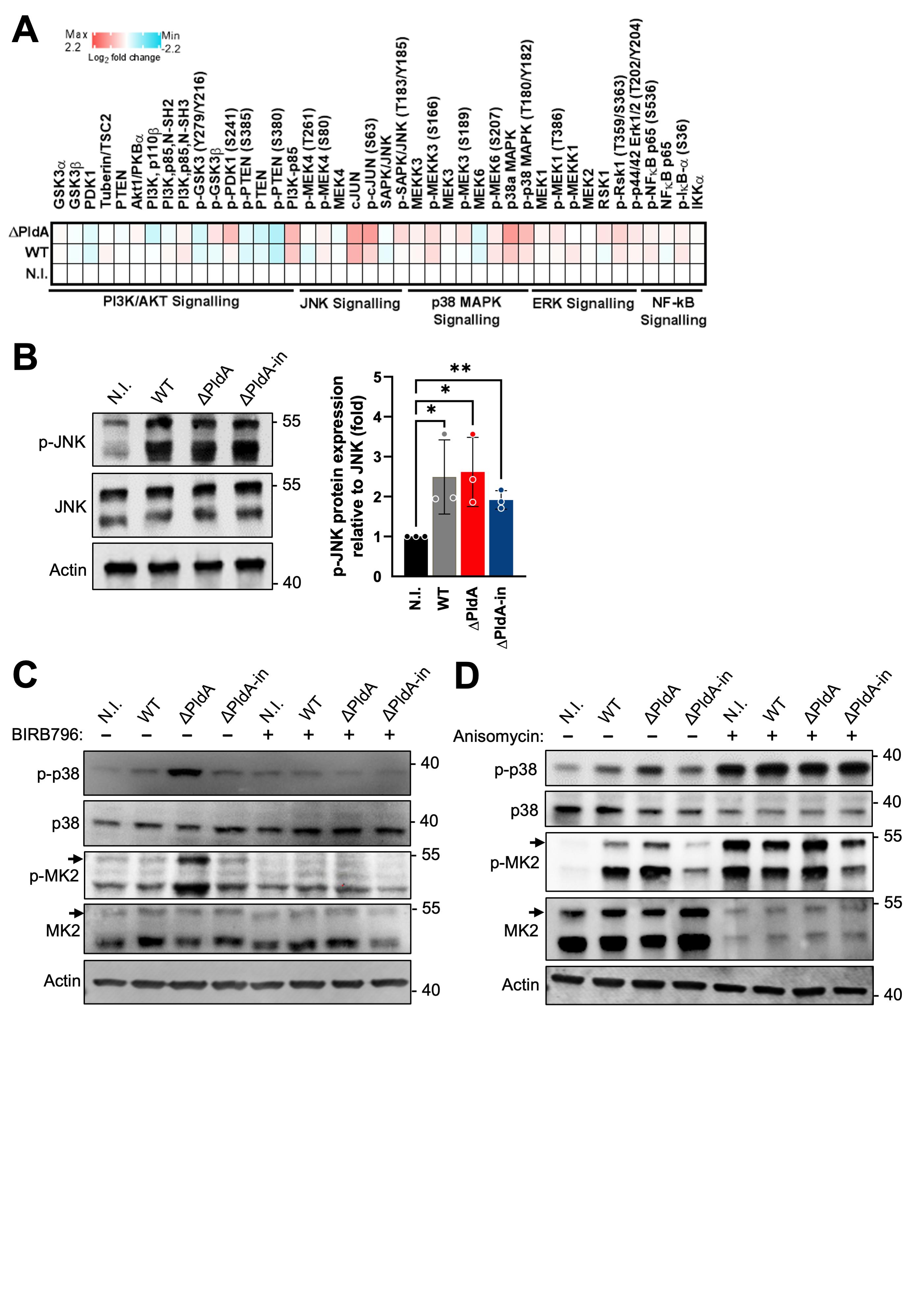

Supplement: Supplemental Material [file KGMI_A_2409924_SM9224.zip › High Res. Figures/High Res. Figures/Figure S5.tiff]

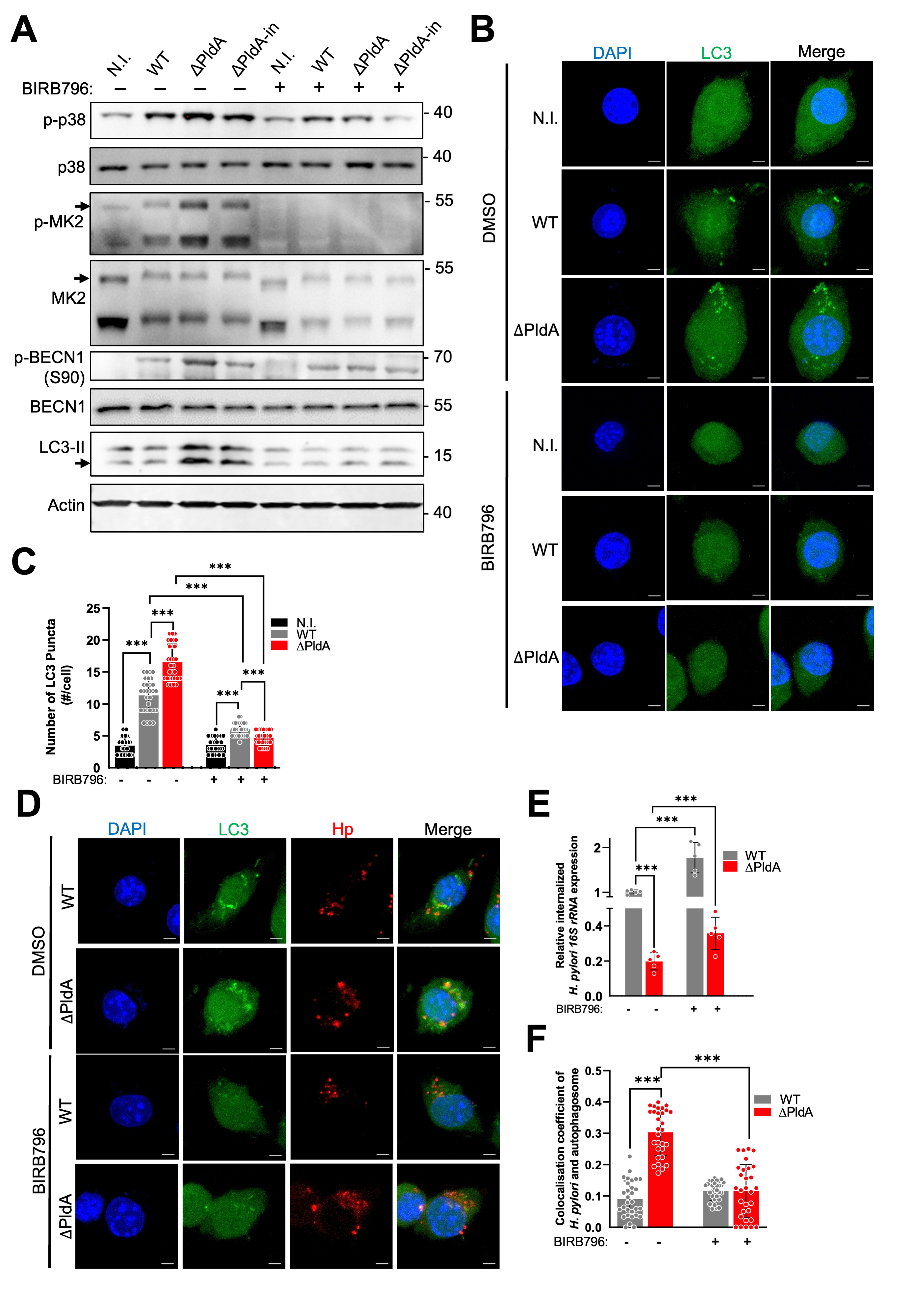

Supplement: Supplemental Material [file KGMI_A_2409924_SM9224.zip › High Res. Figures/High Res. Figures/Figure S6.tiff]

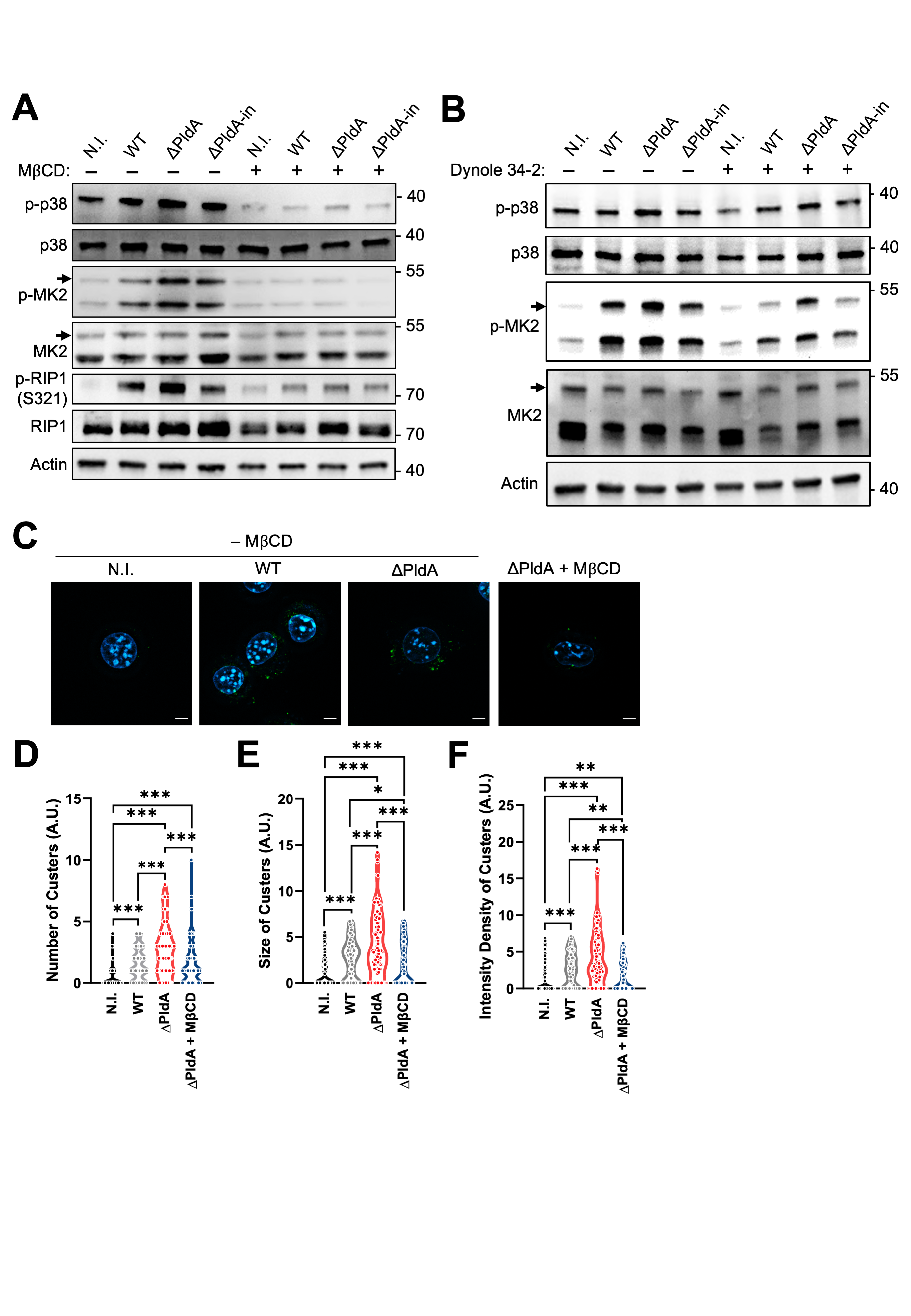

Supplement: Supplemental Material [file KGMI_A_2409924_SM9224.zip › High Res. Figures/High Res. Figures/Figure S7.tiff]

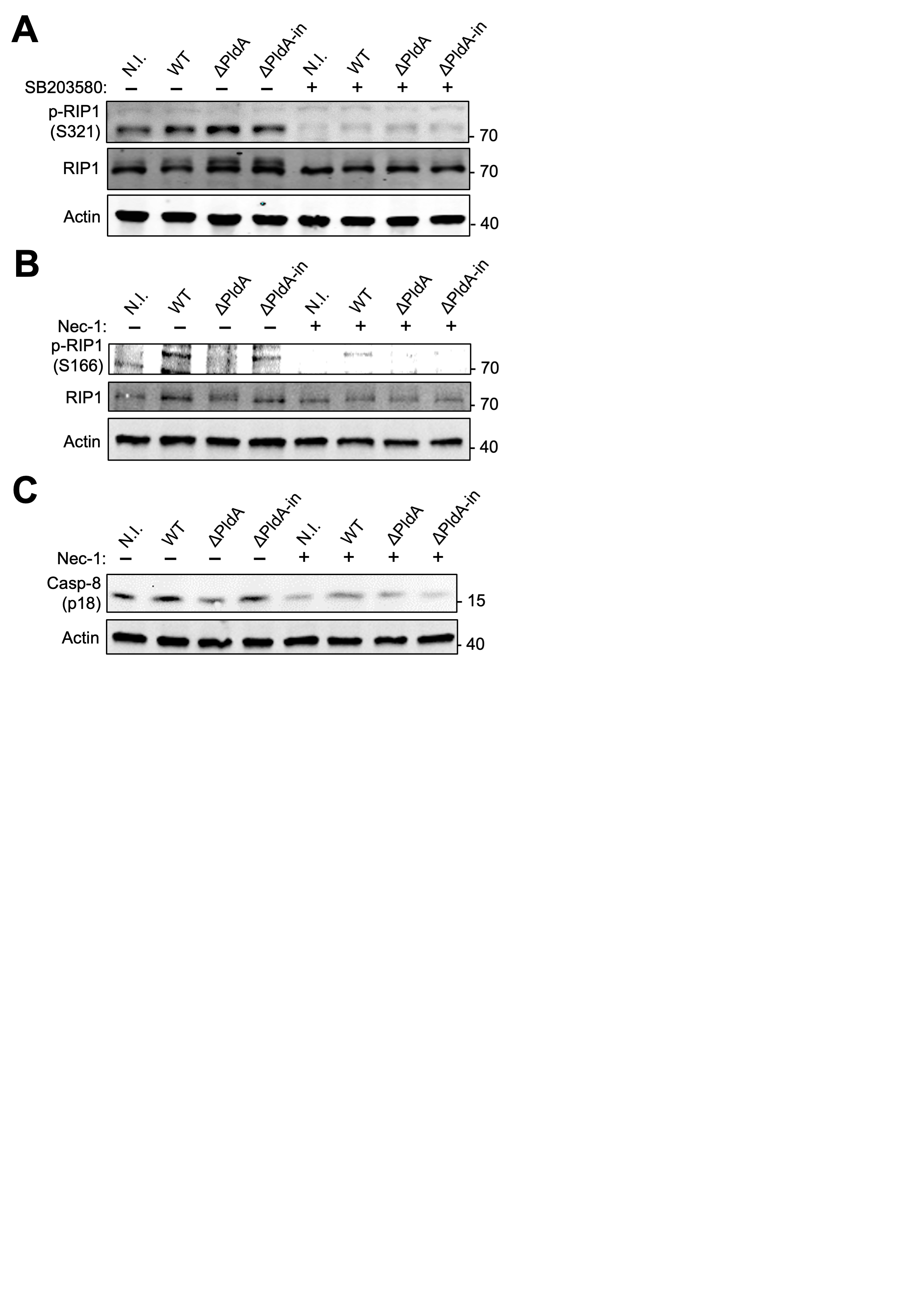

Supplement: Supplemental Material [file KGMI_A_2409924_SM9224.zip › High Res. Figures/High Res. Figures/Figure S8.tiff]

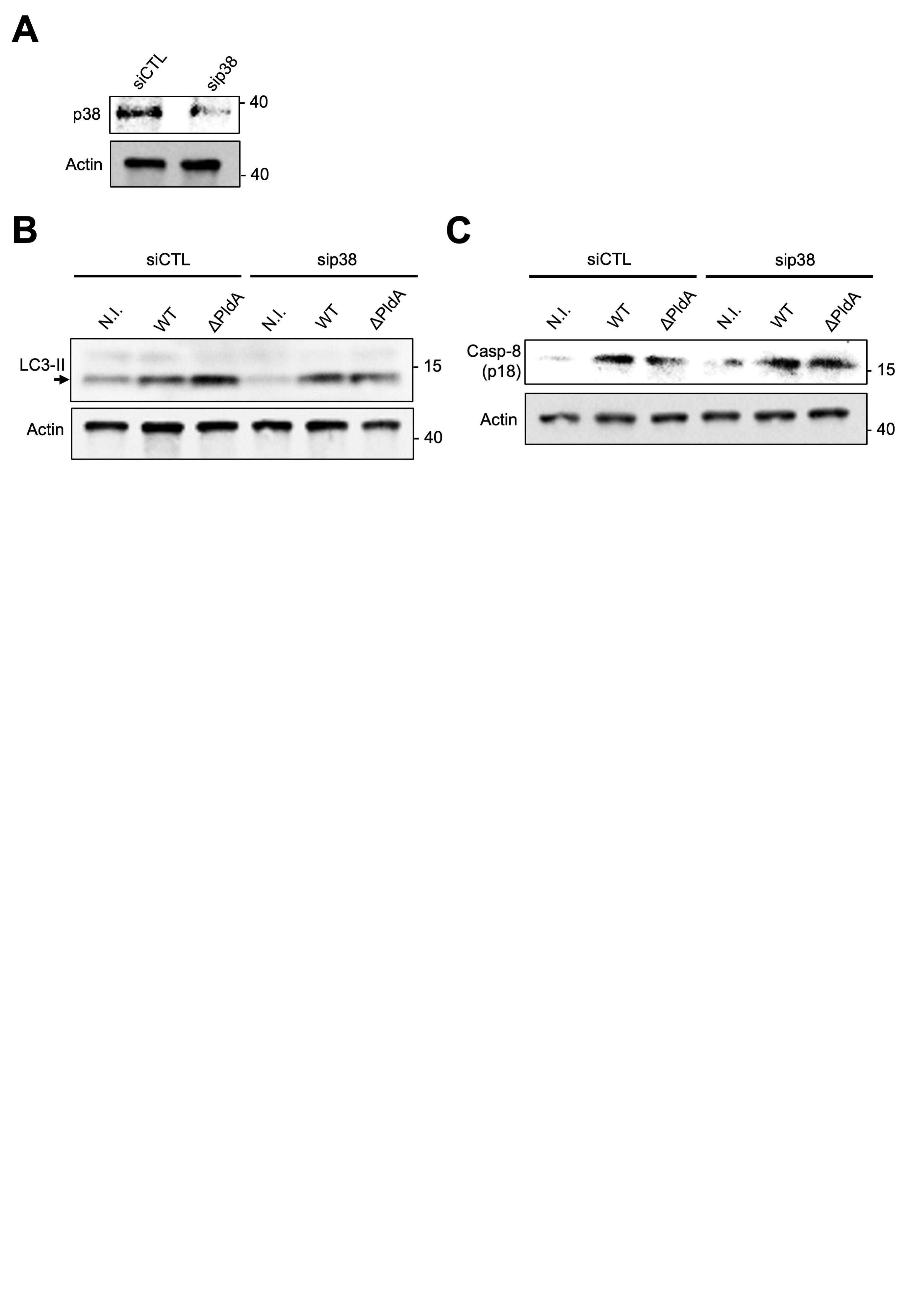

Supplement: Supplemental Material [file KGMI_A_2409924_SM9224.zip › High Res. Figures/High Res. Figures/Figure S9.tiff]
